# Soft matrix promotes ciliogenesis in human retinal pigment epithelial cells

**DOI:** 10.1038/s41598-026-61461-2

**Published:** 2026-07-13

**Authors:** Rida Zahra, Natalie Munding, Katrin Domsch, Hannah Braun-Lee, Camila García-Navarrete, Motomu Tanaka, Gislene Pereira

**Affiliations:** 1 Cytoskeleton, Cell division and Signaling Unit, Centre for Organismal Studies (COS), University of Heidelberg, Heidelberg, Germany; 2 Physical Chemistry of Biosystems, Institute of Physical Chemistry, University of Heidelberg, Heidelberg, Germany; 3 Centre for Organismal Studies (COS), Developmental Biology Unit, University of Heidelberg, Heidelberg, Germany; 4 Present Address: Biomedical Center Munich, Department of Physiological Chemistry, Ludwig-Maximilians-Universität (LMU), Planegg-Martinsried, Munich, Germany; 5 Center for Integrative Medicine and Physics, Kyoto University, Kyoto, 606-8502 Japan; 6 German Cancer Research Centre (DKFZ), DKFZ-ZMBH Alliance, Heidelberg, Germany; 7 Centre for Molecular Biology (ZMBH), University of Heidelberg, Heidelberg, Germany

**Keywords:** Ciliogenesis, Primary cilium, RPE1, Matrix stiffness, Soft matrix, Polyacrylamide hydrogels, Transcriptome, Serum starvation, Autophagy, Atg5, Cell biology, Developmental biology

## Abstract

The primary cilium (PC) is a microtubule-based mechanosensory organelle involved in signal transduction. Although the signaling functions of PC have been well studied, its interaction with the cell´s mechanical environment remains unclear. Here, we used polyacrylamide hydrogels to investigate how extracellular matrix (ECM) stiffness influences ciliogenesis in human retinal pigment epithelial (RPE1) cells. We found that a soft hydrogel (1 kPa) induces cilia formation and elongation in RPE1 cells independent of serum starvation, a classical in vitro method to promote ciliogenesis. Transcriptome analysis of serum-fed RPE1 cells on soft matrix revealed cell cycle exit and alterations in ECM and cytoskeletal gene expression that favor ciliogenesis. Moreover, we observed that despite being very long, cilia formed on a soft substrate are functional, as they transduce Sonic hedgehog signal normally. Transcriptome and microscopy analysis of two cilia-inducing conditions (soft substrate and serum starvation) revealed upregulation of cilia-related genes and downregulation of proliferation markers on both conditions. Autophagy genes were more enriched in serum-starved cells, whereas upregulation of ECM genes and downregulation of actin-related genes were more pronounced on soft matrix. Our study demonstrates that PC biogenesis can be mechanically induced in RPE1 cells on soft substrates independent of external biochemical cues.

**Supplementary Information:**

The online version contains supplementary material available at 10.1038/s41598-026-61461-2.

## Introduction

The primary cilium (PC) is a conserved, non-motile and microtubule-based antenna-like structure which is present in almost all cells in the human body. PC is important to mediate different signaling pathways like Wnt, Sonic hedgehog (SHH), EGFR, NOTCH among others, therefore, it controls many essential biological functions, including cell proliferation, differentiation and tissue homeostasis^1,2^.  Thus, any defect in the process of ciliogenesis could result in various diseases, collectively known as ciliopathies.

Cells form cilia upon exit from the cell cycle when the mother centriole (the older centriole) docks to the plasma membrane and matures into a basal body. Mother centrioles can be distinguished from daughter centrioles due to the presence of distal and subdistal appendages^3^. Distal appendages, radial protrusions at the distal end of mother centrioles, are known to initiate the steps of cilia formation, including the establishment of the ciliary membrane and the elongation of the axoneme (central cilium core, composed of nine doublets of microtubules)^4–6^. Furthermore, intraflagellar transport (IFT) complexes are also important in PC assembly. IFT mechanisms transport protein cargo bidirectionally, i.e., towards and away from the basal body^7^. Tubulin subunits usually enter cilium via IFT mechanisms during axoneme assembly^8^.

PC disassembly is also coordinated with cell-cycle progression. Before re-entering mitosis, cells disassemble the cilium and release the basal bodies, allowing the centrioles to function as microtubule-organizing centers during cell division^9^. PC resorption begins with the depolymerization of axoneme microtubules. Aurora A kinase phosphorylates and activates histone deacetylase HDAC6 which destabilizes axoneme by deacetylating tubulin^10,8^. The balance between PC assembly and disassembly is important for the regulation of PC length^11^. Defects in PC biogenesis and length control mechanisms can affect the development of vital organs^12,13^. Primary cilia are variable in their lengths ranging from 2 μm in chondrocytes to 8–10 μm in neurons^14,15^. These cell type differences suggest that the microenvironment of the cell may play a role in PC length regulation.

Previous reports have shown that extracellular matrix (ECM) components can modify ciliary behavior particularly its length through cytoskeletal modulations^16,17^. For instance, drugs that inhibit microtubules or actin filament formation increase PC length^18–20^. In chondrocytes, direct relationships between the ECM and the PC have been suggested, in part due to the presence of α2, α3, and β1 integrin receptors on chick chondrocyte cilia^21^. Similarly, changes in ECM composition observed in different ciliopathies (e.g., polycystic kidney disease, nephronophthisis) also suggest interactions between the matrix and cilia^17^. These observations raise the question of how cells coordinate matrix-PC signaling and cytoskeleton organization in a growing tissue.

The ECM provides structural support to cells and regulates various cellular processes such as proliferation, migration, and differentiation. ECM stiffness is also important in controlling differentiation, as cells can sense the elasticity of their microenvironment, which in turn influences lineage specification. Soft matrices (0.1–1 kPa) promote neuronal differentiation, whereas stiffer matrices (8–17 kPa) support skeletal muscle lineage, and rigid matrices facilitate the osteogenic differentiation of mesenchymal stem cells^22^, highlighting the tissue-specific functions of the matrix. PC is also involved in the differentiation of stem cells by regulating various signaling pathways^23^. Although both the PC and the ECM control essential cellular functions, including differentiation, the interplay between them in this context remains poorly understood.

In this study, we investigated how ECM stiffness affects ciliogenesis in retinal pigment epithelial cells (RPE1). As a synthetic ECM model, we used chemically crosslinked polyacrylamide hydrogels functionalized with fibronectin, whose stiffness can be flexibly controlled by varying the concentration of the chemical crosslinker^24,25^. We identified an optimal substrate stiffness of 1 kPa that promotes ciliogenesis independent of nutrient deprivation, a common method used to induce cilia in vitro. To understand the global gene profile at 1 kPa that favors ciliogenesis, we performed bulk RNA-seq on serum-rich cells cultured on 1 kPa and glass-control. The findings revealed broad changes on soft substrate related to ECM modulation, adhesion molecules, cell cycle regulation, cilia formation, signaling molecules and cytoskeleton. Because serum depletion is widely used to induce PC in culture, we also analyzed the transcriptome of serum-starved cells grown on uncoated glass relative to the cycling control. Comparison of these two PC-inducing conditions, i.e., mechanical (serum-rich, soft matrix driven) and biochemical (serum starvation based), revealed distinct gene-expression programs, enabling an evaluation of how mechanical regulation and serum deprivation induce cilia biogenesis.

## Results

### Soft substrate increases PC length in serum-starved cells

**Fig. 1:**
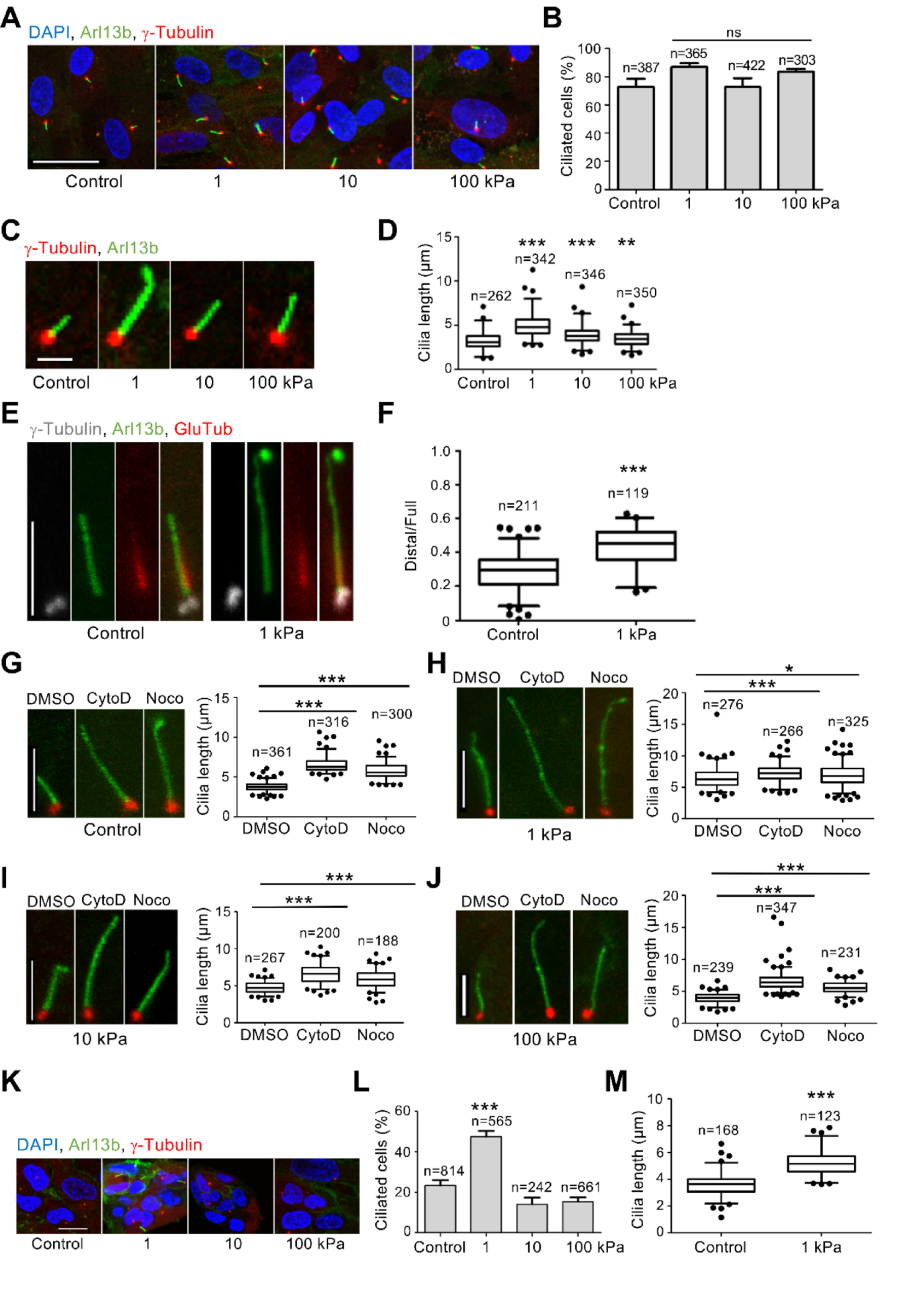
Soft substrate modulates ciliogenesis in serum-fed and starved RPE1 cells. (**A**, **B**) RPE1 *ARL13B-GFP γ-TUBULIN-mRuby2* cells were seeded on glass-control or under different matrix conditions as indicated, and serum starved for 48 h. Cells were inspected by direct fluorescence microscopy after fixation. DAPI served as a nuclear marker. Representative images (**A**) and the percentage of ciliated cells (**B**) are shown. Scale bar, 25 μm. (**C**, **D**) RPE1 *ARL13B-GFP γ-TUBULIN-mRuby2* cells were treated as in (**A**, **B**). Representative images of a cilium (**C**) and quantification of ciliary length (**D**) are shown for the different conditions. Scale bar, 5 μm. (**E**, **F**) RPE1 *ARL13B-GFP γ-TUBULIN-mRuby2* cells were treated as in (**A**, **B**) and additionally stained with glutamylated tubulin antibodies (GluTub). Representative images (**E**) and the ratio of the distal segment to the full cilium length (**F**) are shown. The length of the distal segment was calculated by subtracting the middle segment (GluTub positive) from the full-length cilium (marked by Arl13b). Scale bar, 5 μm. (**G**-**J**) RPE1 *ARL13B-GFP γ-TUBULIN-mRuby2* cells were seeded on glass-control (**G**), 1 kPa (H), 10 kPa (I) or 100 kPa (**J**) matrix samples. Cells were serum-starved for 24 h. Solvent control (DMSO), cytochalasin D (Cyto D) or nocodazole (Noco) was added for 3 h before inspection by direct fluorescence microscopy using fixed samples. Representative images of cilia (red: γ-Tubulin - basal bodies; green: Arl13b - ciliary membrane) and quantifications of ciliary length are shown. Scale bars, 5 μm. (**K**, **M**) RPE1 *ARL13B-GFP γ-TUBULIN-mRuby2* cells were seeded in serum-rich medium on glass-control or under different matrix conditions as indicated, fixed after 24 h of seeding and analyzed by direct fluorescence microscopy. Representative images (K), the percentage of ciliated cells (L) and ciliary length measurements (M) are shown. Scale bar, 25 μm. Data are from two (**A**, **B**) or three (**C**-**M**) independent biological replicates. The sample number (n) is indicated on the graphs. Statistical analysis according to two tailed Mann Whitney test: **, *p* ≤ 0.01; ***, *p* ≤ 0.001; ns, non-significant.

To investigate ciliogenesis, we employed human retinal pigment epithelial (RPE1) cells, a widely used and robust model to study ciliogenesis in culture. The majority of RPE1 cells grown in serum-rich media do not form cilia. However, the percentage of ciliated cells can be increased by switching the cells to serum-free media (serum starvation), a treatment that promotes a G1/G0 cell cycle arrest required for cilia biogenesis^26^. To test the effect of ECM stiffness on PC formation, we used a bis-acrylamide hydrogel-fibronectin system as a synthetic ECM. To promote cell adherence, we coated our gel samples with fibronectin, as it is the main matrix protein reported for RPE1 cells in their natural context^27^. We tested different substrate stiffness, including soft (1 kPa), intermediate (10 kPa) and hard (100 kPa), to find optimal matrix conditions for ciliogenesis (Supplementary Fig. S1A). As control, we used glass coated with fibronectin only (referred to glass-control). To be able to quantify the efficiency of cilia biogenesis and ciliary length, we made use of a previously characterized RPE1 cell line stably producing the basal body marker γ-Tubulin fused to mRuby2, and the cilia membrane protein Arl13b fused to GFP^18^. We did not see any significant difference in the percentage of ciliated cells among these ECM conditions in cells serum-starved for 48 h (Fig. 1A and B). However, cilia were significantly longer in all hydrogel samples compared to the glass-control, with the 1 kPa condition exhibiting significantly longer cilia than the 10 and 100 kPa samples (Fig. 1C and D). Similar results were observed for cells serum-starved for a shorter period (24 h) on 1 kPa-fibronectin-coated hydrogels (Fig. S1B and S1C). Importantly, serum-starved parental RPE1 cells also showed increased ciliary length on 1 kPa substrates both in the absence and presence of serum (Fig. S1D and S1E), indicating that PC elongation on soft matrix is not due to *ARL13B-GFP* or *γ-TUBULIN-mRuby2* expression but a consequence of matrix stiffness. Together, these data suggest that all tested matrix conditions increase ciliary length compared to the fibronectin coated glass-control without interfering with the ability of RPE1 cells to ciliate after serum starvation.

### Soft substrate produces cilia with longer distal segments under serum-starved conditions

We next asked which ciliary domains were extended in cells growing in 1 kPa conditions. We have previously shown that two segments of the axoneme can be differentially elongated depending on the conditions: the middle segment (MS, characterized by enriched polyglutamylation; involved in axoneme stabilization) and the distal segment (DS, low/absent in polyglutamylation; involved in ciliary signaling)^18^. To investigate this, we used polyglutamylated Tubulin as MS marker and compared it with the ciliary membrane marker Arl13b in RPE1 cells stably expressing *γ-TUBULIN-mRuby2* and *ARL13b-GFP*. The length of the DS was calculated by subtracting the MS length from the full-length cilia (marked by Arl13b-GFP). Interestingly, the DS to full-length cilia ratio was significantly higher in 1 kPa condition compared to the glass-control (Fig. 1E and F), indicating that the soft substrate promotes DS elongation.

Cilia elongation is influenced by changes in cytoskeleton organization^19,20^, with cilia becoming longer in conditions that lead to microtubule or actin cytoskeleton depolymerization. As substrate stiffness has been reported to influence cytoskeleton organization^28^, we asked whether cytoskeleton changes could explain the extended cilia phenotype. To address this question, we compared cilia length in cells treated with solvent control, Cytochalasin D (CytoD; actin depolymerization) or Nocodazole (Noco; microtubule depolymerization), cultured on glass-control, 1 kPa, 10 kPa and 100 kPa hydrogels (Fig. 1G and J). In all stiffness conditions, CytoD and Noco treatments further increased the length of cilia compared to solvent control conditions, implying an additive effect and suggesting that changes in cytoskeleton organization alone may not explain the elongated cilia phenotype observed in cells cultured on polyacrylamide hydrogels of different stiffness.

### Soft substrate stimulates PC biogenesis in serum-rich conditions

To explore the effect of matrix stiffness on ciliogenesis in more depth, we asked whether matrix rigidity alone could promote PC biogenesis independent of serum starvation, the established method to induce cilia formation in RPE1 cells. For this, we analyzed RPE1 cells growing in the presence of serum for their ability to ciliate under our three ECM conditions. As expected, a small percentage (20%) of RPE1 cells growing on fibronectin-coated glass were able to ciliate in serum-rich media conditions (Fig. 1K and L). Strikingly, the percentage of ciliation was significantly higher in cells growing on 1 kPa matrix compared to the fibronectin-coated glass-control and other matrix conditions i.e., 10 and 100 kPa (Fig. 1K and L). Cilia at 1 kPa were also significantly longer compared to the control and other matrix conditions (Fig. 1M). These findings indicate that ciliogenesis at 1 kPa is driven by a mechanism that does not depend upon serum starvation.

### Soft substrate downregulates cell cycle progression and upregulates cilia genes in RPE1 cells in serum-rich medium

During the course of our experiments, we observed that nuclei were smaller on 1 kPa substrates in comparison to glass-control (see Fig. S2A-B, DAPI staining, and Fig. S2C, nuclear volume measurements). In agreement with a previous study^29^, we also observed different actin and tubulin organization on soft substrate compared to glass-control or hard substrate in the presence of serum (Fig. S2A, actin; S2B, tubulin). The change in the actin cytoskeleton observed on 1 kPa was not recapitulated when cells were seeded on uncoated glass and serum-starved for 48 h to induce ciliation (Fig. S2D), suggesting that cytoskeleton differences on 1 kPa substrates are primarily associated with substrate stiffness rather than ciliation per se.

**Fig. 2:**
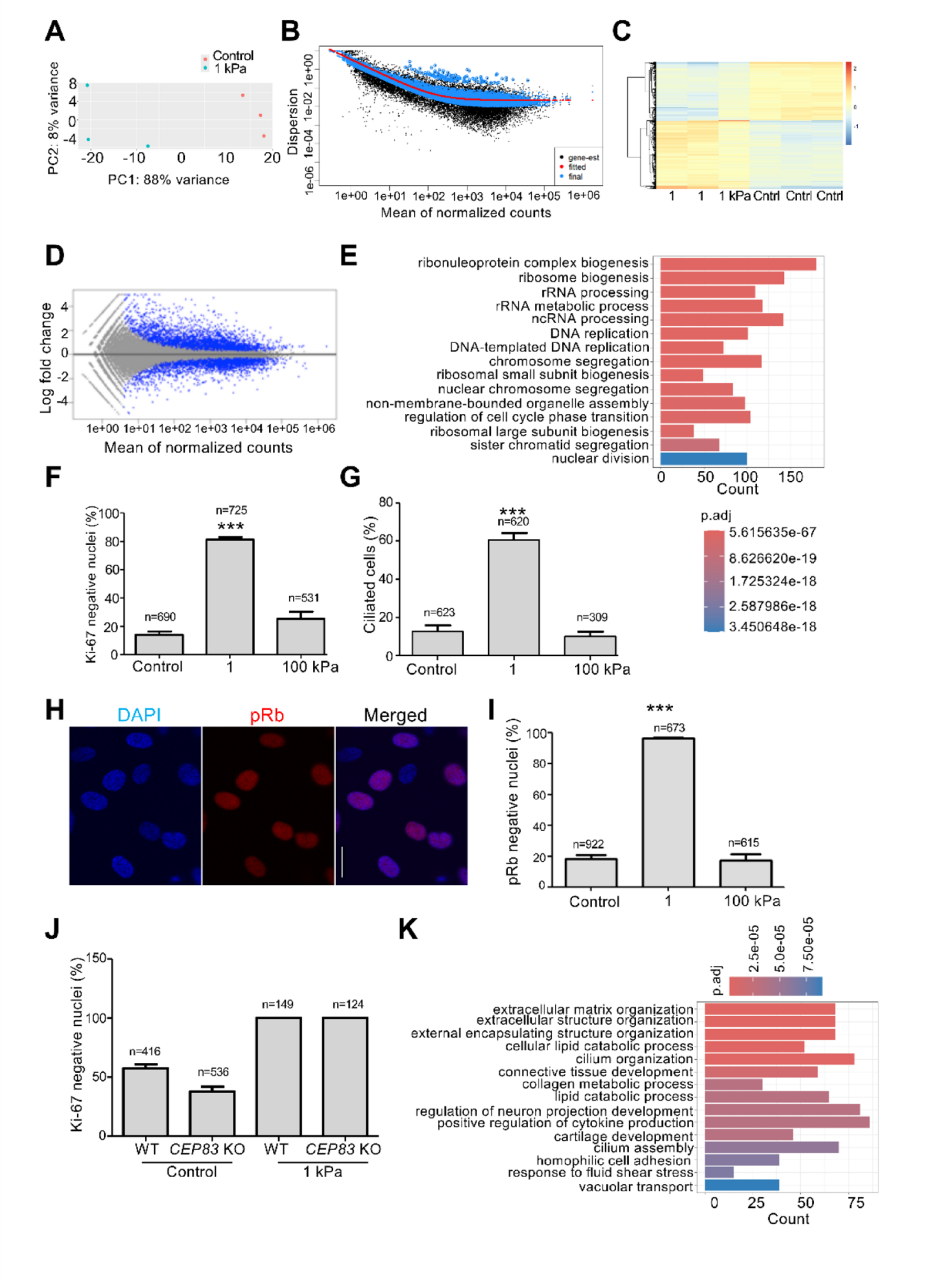
Soft substrate shows cell cycle exit and upregulation of cilia genes. (A-E) Bulk RNA-seq was performed on RPE1 *ARL13B-GFP γ-TUBULIN-mRuby2* cells cultured on 1 kPa and glass-control under serum-rich conditions. (**A**) Principal component analysis (PCA) of control and 1 kPa cells showing a distinct difference between the two conditions. (**B**) Dispersion plot of the transformed data. (**C**) Heatmap of differentially expressed genes (DEG) with *p* < 0.05. (D) MA plot of normalized count and log fold change. (**E**) Gene ontology (GO) analysis depicting the top 15 downregulated biological processes on 1 kPa compared to the glass-control, based on *p* < 0.01 and log2fold change < 0. Data are from three independent biological replicates. (**F**, **G**) RPE1 *ARL13B-GFP γ-TUBULIN-mRuby2* cultured on 1 kPa, 100 kPa and glass-control under serum-rich conditions for 24 h were stained for proliferation markers Ki-67 (**F**). The percentage of ciliated cells (**G**) was quantified based on direct fluorescence microscopy. The percentage of nuclei negative for Ki-67 (F) is shown. Data in (F) are from three independent biological replicates. In (**G**), data shown for 1 kPa and glass-control are from four independent biological replicates, whereas, for 100 kPa, data are from three independent experiments. (**H**-**I**) RPE1-Tet3G cells cultured on glass-control, 1 and 100 kPa in the presence of serum for 24 h were stained for phosphorylated Rb (retinoblastoma). Representative images of pRb staining are shown in (**H**), whereas the complete figure is in supplementary Fig. 5B. Scalebar, 25 μm. The quantification of pRb negative nuclei is shown in (**I**). (**J**) RPE1 wild type (WT) and *CEP83* knockout (KO) cells were cultured on glass-control and 1 kPa matrix in serum-rich media for 4 days. Cells were fixed and stained for Ki-67. The percentage of Ki-67 negative nuclei are shown. Data are from two independent biological replicates. (**K**) GO analysis represents the upregulation of top 15 biological processes on 1 kPa compared to glass-control, based on *p* < 0.01 and log2fold change > 0.25. The sample number (n) is indicated on the graphs. Statistical analysis according to two tailed Mann Whitney test: ***, *p* ≤ 0.001.

We reasoned that morphological differences might lead to differences in the transcriptomic profile of cells growing on hard versus soft matrices. Therefore, to evaluate transcriptomic changes, we performed bulk RNA-seq analysis of RPE1 cells cultured on 1 kPa or fibronectin-coated glass-control under serum-rich conditions. To assess the quality and visualize the distribution of our RNA-seq data, we performed principal component analysis (PCA) and plotted the dispersion plot of the transformed data. PCA showed a clear difference between the expression profile of soft substrate and control (Fig. 2A). Furthermore, the dispersion plot revealed a good fit of gene-wise dispersion (Fig. 2B). The initial analysis revealed 5,773 differentially expressed genes based on *p* < 0.05 (Fig. 2C), the overall distribution is shown as the MA plot indicating 14,910 upregulated and 14,039 downregulated genes (Fig. 2D).

For gene ontology (GO) analysis, we chose more stringent *p value* and log2fold change criteria to filter out upregulated (*p* < 0.01, log2fold change > 0.25) and downregulated (*p* < 0.01, log2fold change < 0) genes. Based on these criteria, we sorted 2,182 upregulated and 1,806 downregulated genes at 1 kPa compared to the control. GO analysis for biological processes (BP) revealed transcriptional down regulation of 259 genes contributing to DNA replication, cell cycle and chromosome segregation in serum-rich 1 kPa samples (Fig. 2E and Fig. S3). Furthermore, GO analysis for molecular function (MF) and cellular components (CC) also confirmed decreased expression of DNA replication and cell cycle-related genes on soft substrate (Fig. S4A and S4B), implying cell cycle exit under serum-rich conditions.

To validate these findings, we determined the proliferation potential of cells cultured on soft substrate under serum conditions using two proliferation markers, Ki-67 and pRb. Ki-67 coats condensed chromosomes in mitosis, forming a perichromosomal layer sheath that prevents chromosomal aggregation^30^; whereas Rb (retinoblastoma) regulates G1/S entry. In its active and hypophosphorylated form it inhibits E2F leading to cell cycle arrest. Phosphorylation of Rb by CDKs (at Ser807/811) results in its inactivation that facilitates G1 to S entry^31^. Therefore, phosphorylated Rb (pRb) and Ki-67 positive cells are considered as dividing cells. We observed that in the presence of serum, contrary to the glass-control and 100 kPa sample, most of the cells at 1 kPa were Ki-67 negative (Fig. 2F and Fig. S5A), which was correlated with higher percentage of ciliated cells on 1 kPa matrix (Fig. 2G). Higher percentage of pRb negative cells was also observed on soft matrix compared to 100 kPa and glass-control under serum-rich conditions (Fig. 2H and I, Fig. S5B). Similarly, the percentage of pRb negative cells significantly increased upon 48 h of serum starvation in comparison to serum-fed cells cultured on uncoated glass (Fig. S5C). Therefore, the vast majority of cells at 1 kPa (serum-rich) or serum-starved (uncoated glass) are in the non-proliferative state.

As cilia have been proposed to put a brake on the cell cycle^32,33^, we hypothesized that the non-proliferative state of RPE1 cells on 1 kPa substrates might be dictated by the presence of cilia. To test this, we used RPE1 *CEP83* knockout (KO) cells. These cells are unable to ciliate, as Cep83 is a distal appendage component required for ciliogenesis (Fig. S5E)^34^. The analysis revealed that *CEP83* KO cells, similar to the WT control, remained in a non-proliferative state on 1 kPa substrates, even after four days of culturing them in serum (Fig. 2J and Fig. S5D). We thus concluded that a soft substrate of 1 kPa promotes a non-proliferative state in RPE1 cells independent of cilia.

**Fig. 3:**
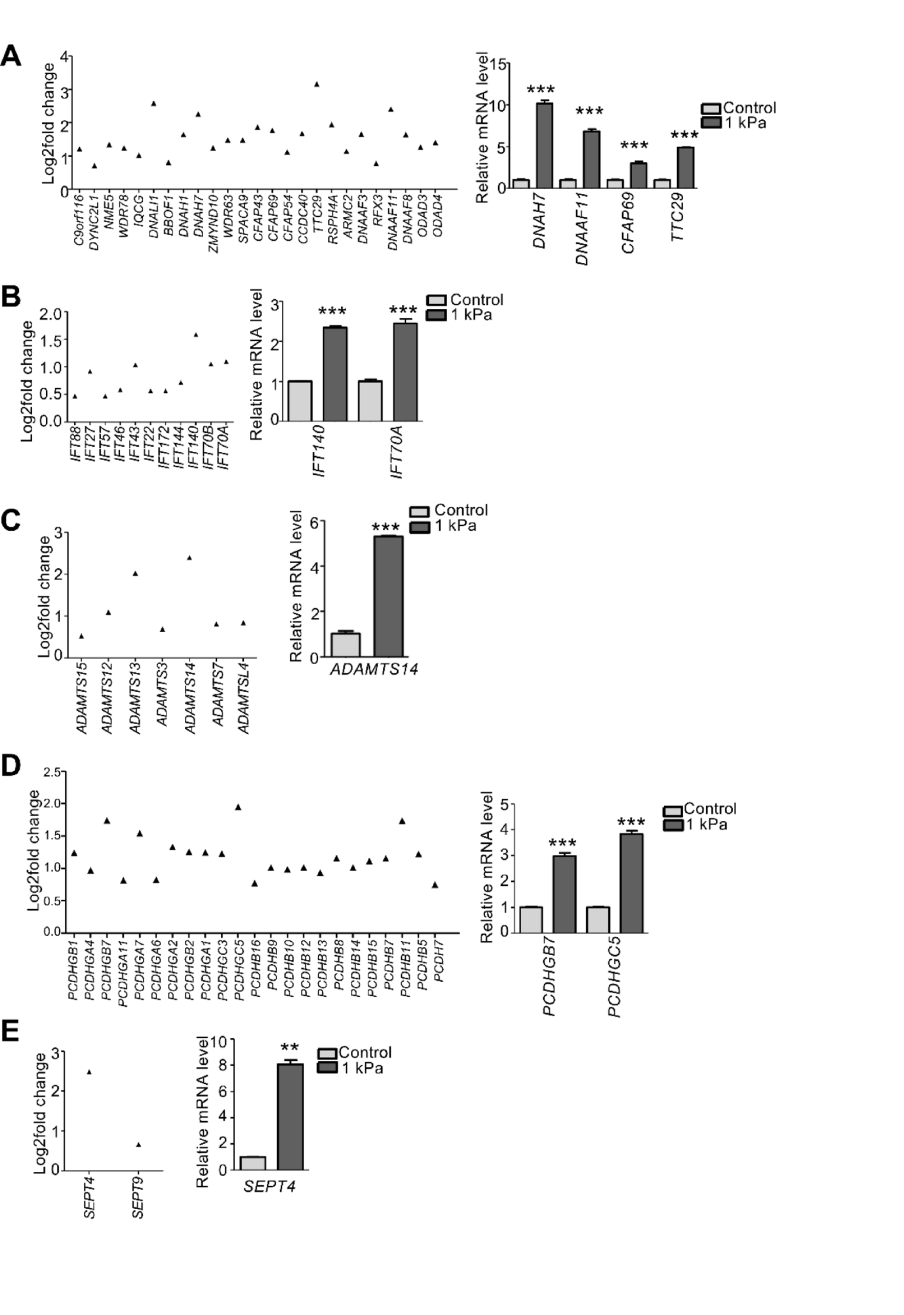
Real time (RT) PCR confirms differential transcriptomic profile at 1 kPa. (**A**-**E**) Log2fold change of genes found to be upregulated on 1 kPa matrix by bulk RNA-seq analysis (graphs to the left) and relative mRNA levels validated by RT-PCR (graphs to the right) are shown for ciliary motility genes (**A**), IFT transport genes (**B**), matrix ADAM proteases (**C**), protocadherins (**D**) and septins (**E**). Data are from three independent biological replicates, except for IFT70A (RT-PCR analysis - two biological replicates).

In line with increased cilia formation, 78 genes (out of 2,182 upregulated genes) associated with the GO terms “cilium assembly” and “cilium organization” (Fig. 2K) were upregulated on 1 kPa substrates compared to the glass-control. These included genes related to axoneme transport (e.g. *IFT140*, *IFT43*, *IFT22*, *IFT172* etc.), centrosome function (*CEP19*, *FAM161B*, *CEP126*, *CBY1*), ciliary vesicles (*RAB17*, *EHD3*, *RILP*) and axoneme length and stability (*SPEF1*, *RP1*) (Fig. S6A). In agreement with the critical role of intraflagellar transport in PC formation and elongation^35,7,36^, we observed decreased ciliation on soft substrates under serum-rich conditions upon *IFT88* depletion (Fig. S6B). Surprisingly, we also observed that 26 genes related to ciliary motility, including dyneins (*DNALI1*, *DNAH1*, *DNAH7*, *DNAAF3*, *DNAAF11*, *DNAAF8* among others) and cilia/flagella-associated proteins (CFAPs: *CFAP43*, *CFAP54*, *CFAP69*, *CFAP263*), were upregulated on 1 kPa (Fig. S6A and Fig. 3A). The upregulation of a sub-set of ciliary motility genes (*DNAH7*, *DNAAF11*, *CFAP69* and *TTC29*) as well as axoneme transport (*IFT140* and *IFT70A*) was further confirmed by real time (RT)-PCR (Fig. 3A and B).

These findings together indicate that the soft ECM condition changes RPE1 transcriptome in a way that favors PC formation by inhibiting the cell cycle despite the presence of serum.

### Soft substrate modulates the transcriptome of ECM, cell adhesion and cytoskeleton related genes

Out of 2,182 upregulated genes, 68 genes related to the GO term “extracellular matrix organization”, including matrix proteases (*MMP15*, *MMP11*, *MMP19*, *ADAMTS14*, *ADAMTS15* and others), collagen (*COLQ*, *COL9A3*, *COL6A1*, *COL14A1* and others) and laminin genes (*LAMB1*, *LAMC1*), were upregulated in cells growing on 1 kPa compared to the glass-control (Fig. S6C). The upregulation of the ADAM protease *ADAMTS14* was also confirmed by RT-PCR (Fig. 3C), depicting that substrate stiffness modulates the ECM transcriptome.

As cells growing on 1 kPa formed dense colonies that did not spread, we reasoned that adhesion molecules might also be upregulated on soft substrates. GO analysis indicated increased expression of 39 genes related to “homophilic cell adhesion via plasma membrane adhesion molecules“ (Fig. S7A), which included upregulation of 23 protocadherins (*PCDHGB1*, *PCDHGB7*, *PCDHGA4*, *PCDHGA7*, *PCDHGC5* among others) and two cadherins (*CDH13*, *CDH18*) on soft substrate compared to glass-control. The upregulation of two protocadherins, *PCDHGB7* and *PCDHGC5*, was also validated by RT-PCR (Fig. 3D). The enhanced expression of adhesion molecules might account for high cell-cell contact, colony formation and less migration of RPE1 cells on soft gels.

Out of 1,806 downregulated genes, 30 genes were related to the GO-term “actin filament bundles”, including genes involved in focal adhesion (*TLN2* and *VCL*), actin polymerization (*ACTR3* and *ACTR2*), actin nucleation (*DIAPH1* and *DIAPH3*) as well as myosin related genes (*MYO19*, *MYO7B*, *MYO1C*, *MYH9*) (Fig. S7B). This indicates differential expression of the cytoskeletal genes in 1 kPa that might impinge upon differences in cytoskeleton organization observed in cells growing on soft substrates. Contrary to the actin genes, two septins (cytoskeleton associated proteins), *SEPT4* and *SEPT9*, were upregulated on soft substrate (Fig. 3E). Our RT-PCR results also supported the upregulation of *SEPT4* on soft matrix (Fig. 3E). In summary, these observations suggest that soft ECM changes cell behavior and morphology by upregulating adhesion molecules and changing cytoskeleton genes at the transcriptional level.

### Soft substrate increases *GLI1* expression and changes YAP/TAZ nuclear to cytoplasmic ratio

**Fig. 4:**
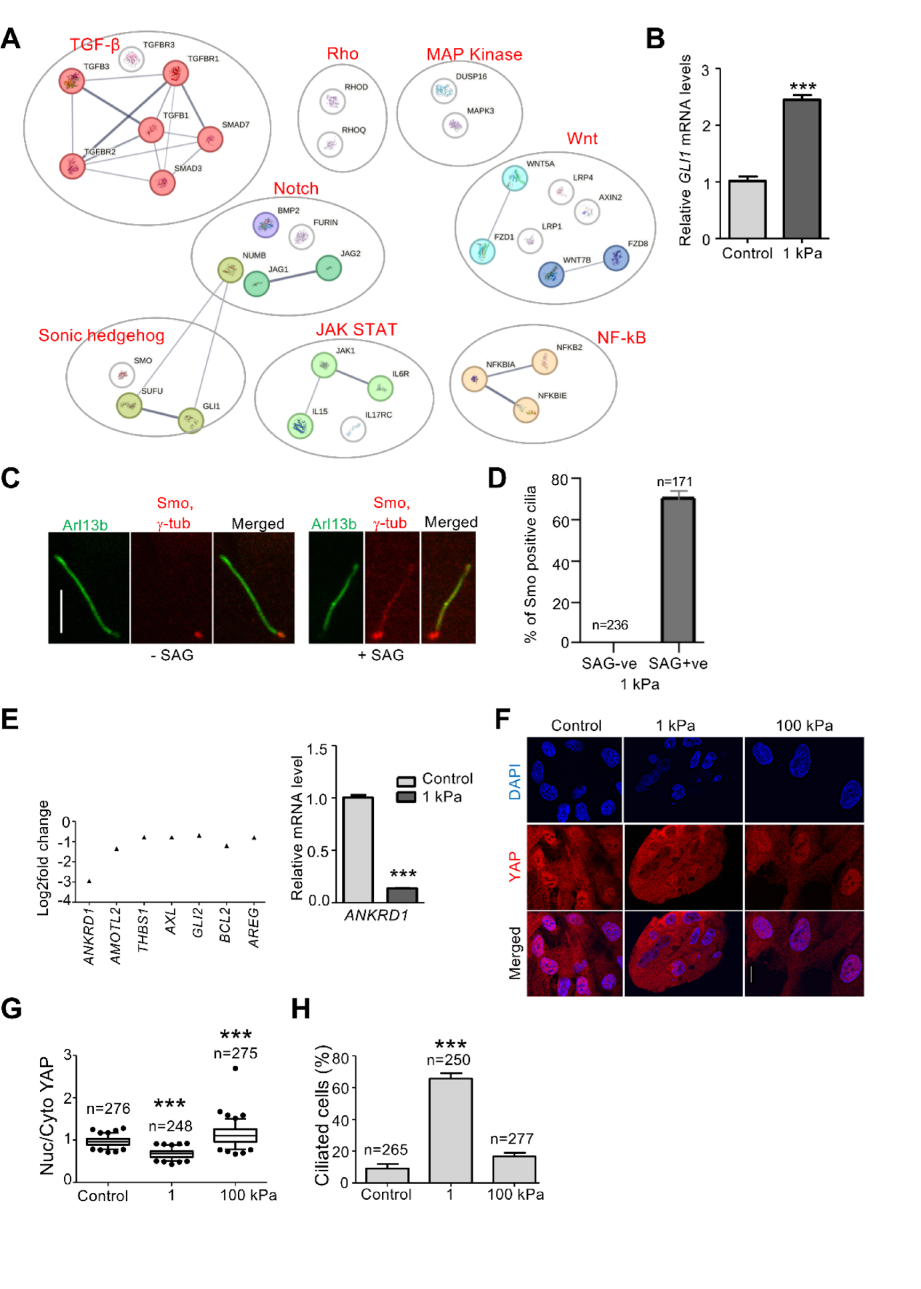
Soft substrate regulates signaling pathways. (**A**) Components of signaling pathways found to be upregulated on 1 kPa matrices by bulk RNA-seq analysis. (**B**) Relative mRNA levels of *GLI1*, determined by RT-PCR in RPE1 *ARL13B-GFP γ-TUBULIN-mRuby2* cells cultured on glass or 1 kPa matrices in serum-rich media for 24 h. Data are from three independent biological replicates. (**C**, **D**) Smo cilia localization in RPE1 *ARL13B-GFP γ-TUBULIN-mRuby2* cells cultured on 1 kPa matrices in serum-rich media for 24 h, followed by an additional 24 h incubation in the absence (- SAG) or presence (+ SAG) of the Smoothened agonist SAG (200 nM). Smo was detected by indirect fluorescence microscopy using anti-Smo antibodies. The basal bodies (red) and ciliary membrane (green) were visualized by direct fluorescence. Representative images (**C**) and quantification of the percentage of Smo-positive cilia (**D**) are shown. Data from two independent biological replicates. Scale bar, 5 μm in (**C**). (**E**) Scatter plot showing the reduced expression of YAP target genes on 1 kPa matrices based on bulk RNA-seq analysis (graph on the left) and relative mRNA levels of the YAP target *ANKRD1* determined by RT-PCR (graph on the right). Data from three independent biological replicates. (**F–H**) Analysis of YAP localization in RPE1 *ARL13B-GFP γ-TUBULIN-mRuby2* cells cultured on glass-control, 1 kPa and 100 kPa matrices in serum-rich media for 24 h. YAP was stained using anti-YAP antibodies. Representative images (**F**), nuclear to cytoplasm YAP ratios (**G**) and percentage of ciliated cells ((**H**), determined by direct fluorescence) are shown. Scale bar, 10 μm. Data from three independent biological replicates. The sample number (n) is indicated on the graphs. Statistical analysis according to two tailed Mann Whitney test: ***, *p* ≤ 0.001.

We found that components of various pathways, including MAP kinase, NF-kB, TGF-β and SHH, were transcriptionally upregulated on soft substrate (Fig. 4A). In line with PC acting as a signaling hub for SHH^37,38^, we asked whether SHH pathway was constitutively active on 1 kPa. Pathway activation in ciliated cells (PC formed upon serum starvation on glass-control) initiates with the entrance of the SHH regulator Smoothened (Smo) into the cilium upon SAG treatment (Smoothened agonist) (Fig. S8A), where it initiates a cascade of events, eventually leading to transcriptional activation, including the transcriptional upregulation of *GLI1* transcription factor^39^. On 1 kPa substrates in the absence of SAG, we confirmed that *GLI1* mRNA levels were higher in comparison to glass-control (Fig. 4B). However, under this condition, Smo did not accumulate in the cilium (Fig. 4C and D). Smo cilia accumulation was only considerably higher at 1 kPa in the presence of SAG (Fig. 4D).

In addition to the above mentioned signaling components, we also analyzed YAP (Yes associated protein), as it has been reported to respond to mechanical cues^40^. Transcriptional activator YAP and its homolog TAZ shuttle between cytoplasm and nucleus in a confluency dependent manner^41^. Contrary to the glass-control, cells at 1 kPa were densely packed in the form of colonies and they did not spread on the substrate in the presence of serum. Dense cellular colonies correlate with YAP/TAZ inactivation and cytoplasmic accumulation^42^. We found that YAP transcriptional target genes (*ANKRD1*, *AMOTL2*, *THBS1*, *AXL*, *GLI2*, *BCL2* and *AREG*) were downregulated in our dataset (Fig. 4E). RT-PCR confirmed the downregulation of the YAP target, *ANKRD1* (Fig. 4E).

High cell density had been reported to induce cilia formation through inactivation and cytoplasmic accumulation of YAP/TAZ^43^. To investigate whether YAP/TAZ would also become more cytoplasmic in soft substrates, we determined the nuclear to cytoplasmic ratio of YAP in cells cultured on hard (glass-control and 100 kPa) and soft (1 kPa) substrates under serum-rich conditions. YAP was more nuclear in glass-control and 100 kPa samples, whereas it was more cytoplasmic in 1 kPa samples (Fig. 4F and G). YAP inactivation coincided with increased ciliation rate on soft matrix under serum-rich conditions (Fig. 4H). These observations indicate that YAP/TAZ inactivation might contribute to ciliogenesis on soft substrates. In contrast, in our 48 h serum-starved condition (cells cultured on uncoated glass) of PC induction, YAP nuclear to cytoplasmic ratio was higher (Fig. S8B, median nuclear to cytoplasmic ratio of 1.2) than the 1 kPa condition (Fig. 4G, median nuclear to cytoplasmic ratio of 0.7), suggesting that nutrient deprivation-driven cilia formation occurs even though YAP remains relatively more nuclear, while still being within the range previously described as non-nuclear and permissive for ciliogenesis (nuclear to cytoplasmic ratio of 1.2 or less)^44^.

### Transcriptional profile of RPE1 cells upon serum starvation indicates upregulation of autophagy and cilia-related genes

We next asked how the transcriptome of our RPE1 cell line changes when the cells are subjected to serum starvation, a treatment frequently used to induce ciliation in RPE1 cells. To address this question, we incubated RPE1 cells (cultured on uncoated glass) for 6 h and 24 h in the presence or absence of serum before processing the samples for bulk RNA-seq analysis. Early steps of cilia formation, including trafficking of proteins to the centrosome, was shown to initiate shortly after serum starvation in RPE1 cells^45,46^, yet only a small percentage of fully formed cilia were observed after 6 h of serum starvation (17%). In contrast, approximately 50% of the cells showed a fully elongated cilium after 24 h of starvation. We therefore anticipated that cellular responses to serum starvation would be more prominent at 6 h in serum-free media, whereas cilia-related specific transcriptional changes may become more apparent after 24 h. PCA, MA plot, dispersion plot and heatmaps of the transcriptomic data indicated no systematic bias and a clear difference between control and experimental conditions (Fig. S9A-S9F). When considering a log2fold change > 0.25 and *p* < 0.01, 30% (4,920 out of 15,733) and 16% (4,689 out of 29,481) of the hits were upregulated in cells serum-starved for 6 h and 24 h, respectively, compared to their corresponding controls. To identify downregulated genes, we considered a log2fold change of < 0 and *p* < 0.01. Under these criteria, 2,675 and 4,822 genes were downregulated in 6 h and 24 h of serum starvation, respectively, compared to controls.

**Fig. 5:**
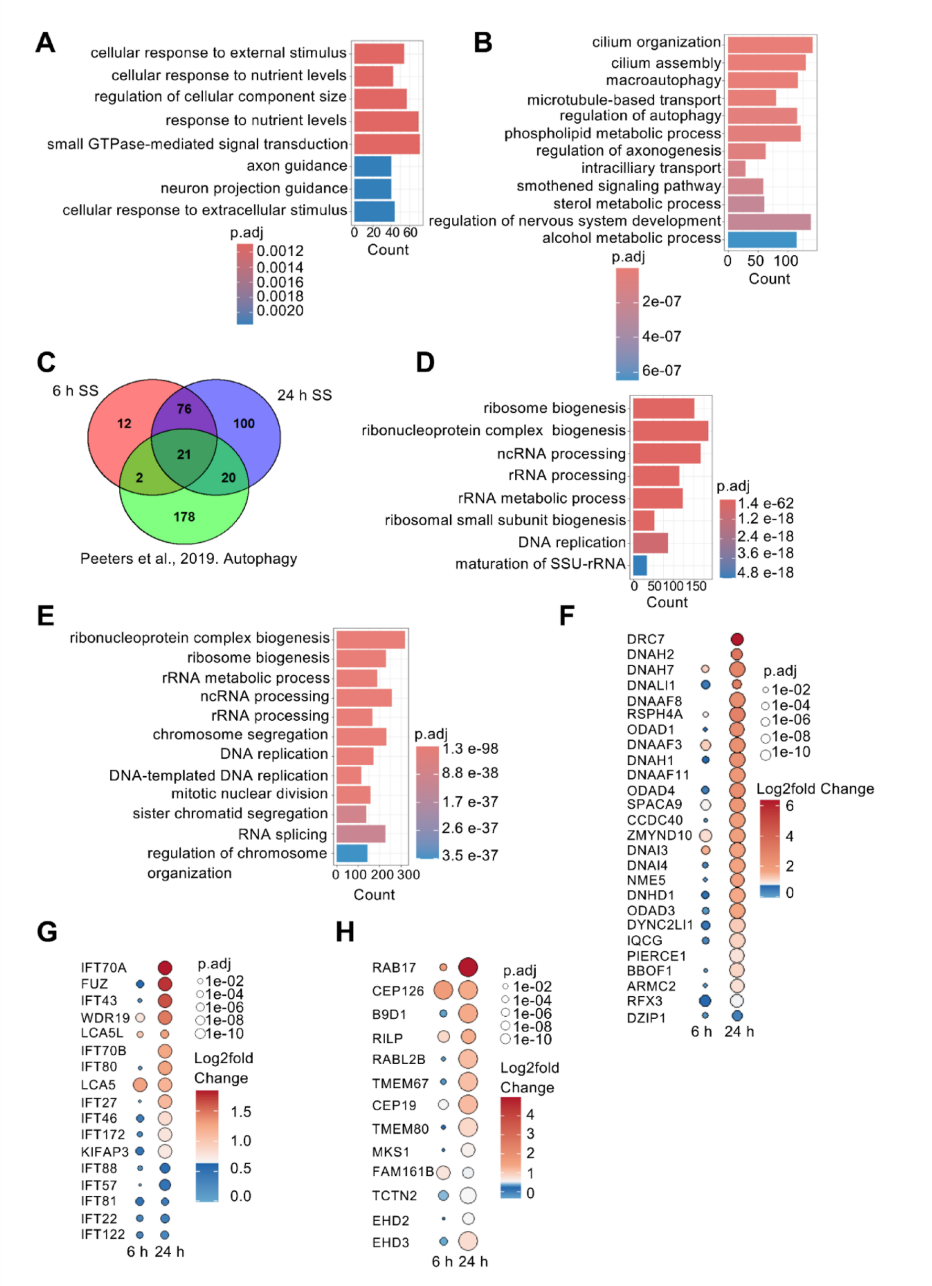
Gene ontology (GO) analysis shows prominent upregulation of cilia genes after 24 h of starvation. RPE1 *ARL13B-GFP γ-TUBULIN-mRuby2* cells seeded on uncoated glass were subjected to bulk RNA-seq after 6 h and 24 h of culture in serum-rich (control) or serum-free (serum starved) medium. (**A**, **B**) Gene ontology analysis showing upregulated biological processes at 6 h (**A**) and 24 h (**B**) of serum starvation compared to controls (serum-rich conditions), based on *p* < 0.01 and log2fold change > 0.25. (**C**) Venn diagram showing the number of common and unique upregulated autophagy-related genes identified in RPE1 *ARL13B-GFP γ-TUBULIN-mRuby2* cells after 6 h and 24 h of serum starvation, compared to the previous study by^48^ using human HAP1 cells serum starved for 6 h. (**D**, **E**) GO analysis showing downregulated biological processes at 6 h (**D**) and 24 h (**E**) SS, based on *p* < 0.01 and log2fold change < 0. (**F**, **H**) Bubble plots showing the expression of ciliary motility genes (**F**), axoneme transport genes (**G**) and genes related to centrosome, ciliary vesicle trafficking and ciliary transition zone (**H**) after 6 h and 24 h of serum starvation compared to their respective controls.

Cellular response to serum starvation was shown to involve the upregulation of genes related to autophagy and downregulation of genes related to cell cycle^47,48^. Accordingly, we found that after 6 h in serum-free medium, genes related to cellular response to starvation, including biosynthetic processes and autophagy-related genes, were significantly upregulated (Fig. 5A). The number of upregulated autophagy genes compared to the control also increased significantly after 24 h of serum starvation (Fig. 5B and C). Furthermore, we observed an overlap of these genes with a previous study using human HAP1 cells^48^(Fig. 5C). To confirm that autophagy activation was indeed increased upon serum starvation, we analyzed RPE1 cells stably expressing the mCherry-GFP-LC3 reporter, in which the autophagy adapter protein LC3 (microtubule-associated protein 1 light chain 3) is fused to mCherry and GFP tandem tags^49,50^. Due to instability of GFP fluorescence at acidic pH, the mCherry-GFP-LC3 reporter gives a yellow signal at the cytoplasm and autophagosomes (mCherry+, GFP+ puncta) but only a red signal at autolysosomes (mCherry+, GFP- puncta), allowing us to estimate the autophagic flux (represented by the ratio of red to yellow foci per cell). As expected, the autophagic flux was significantly increased in serum-starved cells (Fig. S10).

A subset of 81 DNA-replication genes was downregulated at 6 h of serum starvation (Fig. 5D). We further observed significant enrichment of downregulated cell cycle genes after 24 h of starvation (230 more genes compared to 6 h of starvation, including *CDK1*, *CDC27*, *CDC20*, *CDC14A*, *AURKB*, and *PLK1*) (Fig. 5E). These findings were consistent with the higher percentage of non-dividing RPE1 cells in cultures serum-starved for 24 h (73.5%) compared to 6 h (11.9%), as determined by pRb analysis. Furthermore, we observed a higher proportion of upregulated cilia-related components at 24 h than at 6 h of serum starvation, when compared with their respective controls (Fig. 5F-H). This confirms that ciliary-related responses to starvation begin shortly after nutrient deprivation and become more pronounced by 24 h, correlating with a higher percentage of ciliated cells.

### Comparison of the transcriptome landscape of serum-starved and 1 kPa cilia-inducing conditions

**Fig. 6:**
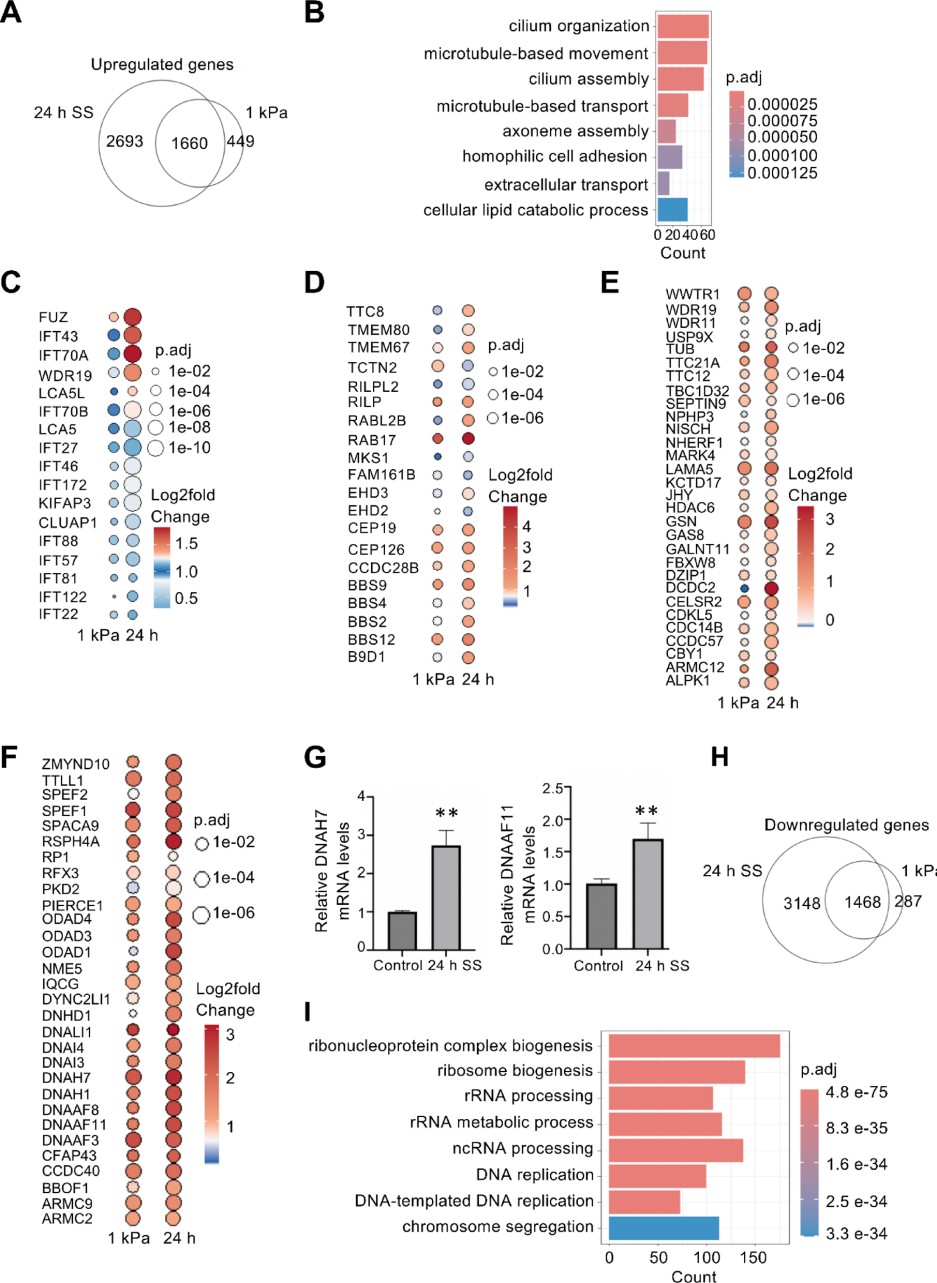
Overlap of gene expression programs induced by 1 kPa matrix and serum starvation. (**A**) Venn diagram showing the number of genes upregulated on soft matrix (1 kPa) relative to the glass-control, and after 24 h of serum starvation relative to serum-rich conditions. (**B**) Gene ontology analysis showing biological processes enriched among genes commonly upregulated on both 1 kPa and 24 h serum starvation conditions. (**C**-**F**) Bubble plots showing the expression of upregulated cilia-related genes associated with axoneme transport (**C**), centrosome, BBSome, cilia vesicle trafficking and ciliary transition zone (**D**), other cilia-related processes (**E**), and ciliary motility and axoneme stability (**F**) on 1 kPa and 24 h serum-starved conditions relative to their respective controls. (**G**) Relative mRNA levels of *DNAH7* and determined by RT-PCR after 24 h of serum starvation (SS) compared with serum-rich condition (control). Data are from three independent biological replicates. **, *p* ≤ 0.01. (**H**) Venn diagram depicting the number of genes downregulated on 1 kPa relative to the glass-control, and after 24 h of serum starvation compared with serum-fed cells. (**I**) Gene ontology analysis showing biological processes commonly downregulated on both 1 kPa and 24 h of serum starvation.

Next, we compared the transcriptome landscape of both cilia inducing conditions, i.e., mechanical condition (induced by a soft substrate in the presence of serum) and biochemical condition (induced by serum starvation on uncoated glass). We hypothesized that a common set of genes related to cilia induction would be shared between both conditions, while alternative pathways related to matrix stiffness or serum starvation would differ. To investigate these changes, we compared transcriptomic datasets obtained from these two independent conditions that induced ciliogenesis: culture on 1 kPa (in the presence of serum) and after 24 h of serum starvation. Compared to their respective controls, 1,660 genes were commonly upregulated in both conditions (Fig. 6A) (*p* < 0.01 and log2fold change > 0.25). Genes involved in cilia organization, axoneme assembly, and microtubule-based transport comprised the top category among these commonly upregulated genes, with 100 cilia hits (Fig. 6B). Among these, many components of the IFT machinery (IFT-A components: *IFT43*, *IFT122*, *WDR19*. IFT-B components: *IFT70*, *IFT27*, *IFT46*, *IFT172*, *IFT88*, *IFT57*, *IFT81*, *IFT22* and *CLUAP*) and the BBSome complex (*BBS2*, *BBS4*, *BBS9* and *TTC8*) were represented (Fig. 6C and E). Interestingly, although RPE1 cells typically form a primary (immotile) cilium, both conditions showed increased expression of genes related to ciliary motility, with two of them (*DNAH7* and *DNAAF11*) also validated by RT-PCR in 24 h serum-starved cells (Fig. 6F and G). Commonly downregulated genes comprised 1,468 hits (Fig. 6H), with a large representation of genes involved in DNA replication and chromosome segregation (Fig. 6I) (*p* < 0.01 and log2fold change < 0). These transcriptomic changes align with the observed cell cycle exit and increased ciliogenesis under both conditions.

**Fig. 7:**
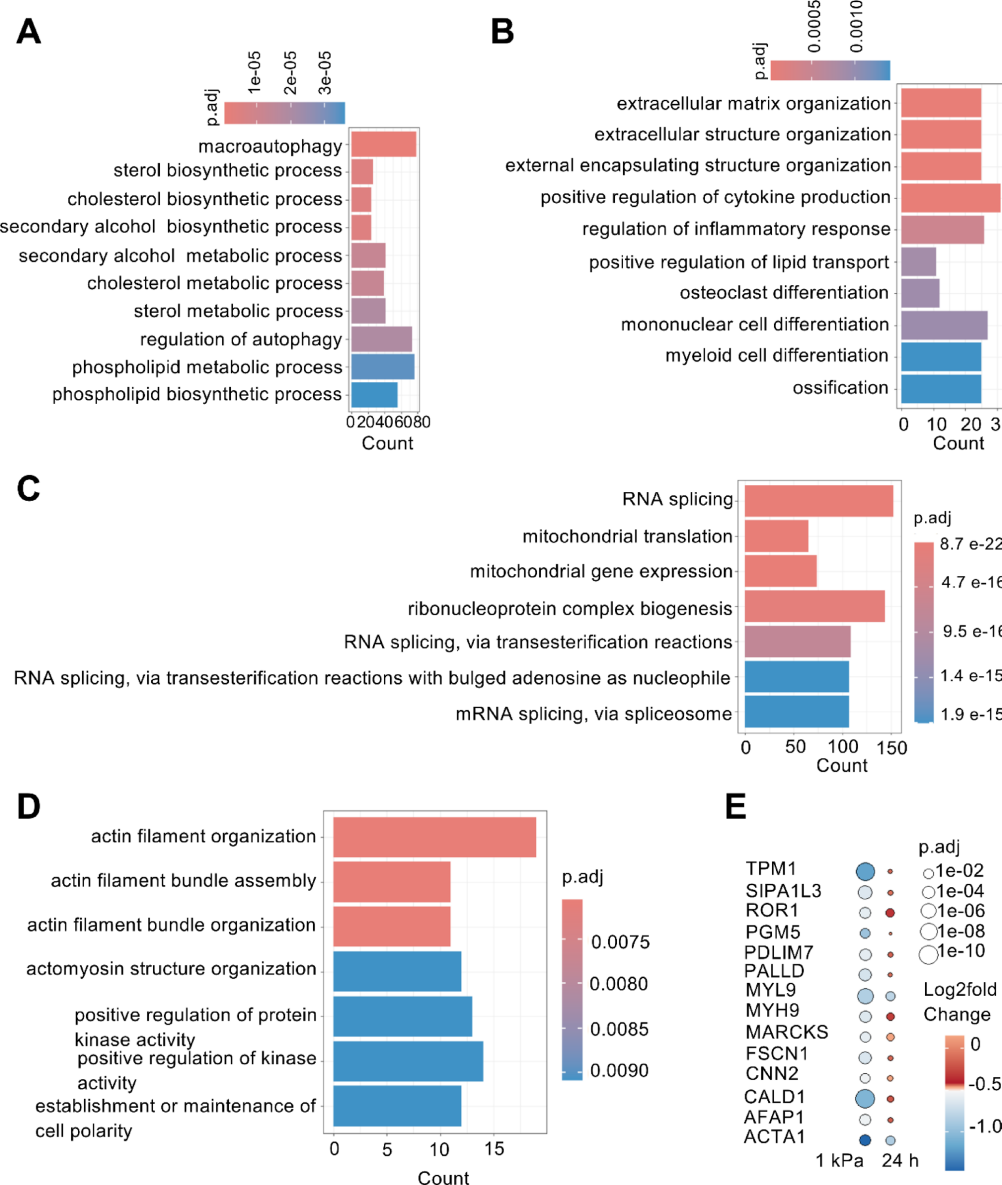
Unique GO terms identified under 1 kPa and 24 h of serum starvation conditions. (**A**, **B**) GO analysis showing biological processes for genes upregulated only after 24 h of serum starvation (**A**) or only under 1 kPa conditions (**B**). (**C**, **D**) GO analysis showing biological processes for genes downregulated only after 24 h of serum starvation (**C**) or only under 1 kPa conditions (**D**). (**E**) Bubble plots showing actin-related genes downregulated under 1 kPa but not after 24 h of serum starvation.

Condition-specific changes were apparent, with 2,693 genes upregulated and 3,148 downregulated exclusively under 24 h serum starvation, and 449 upregulated and 287 downregulated uniquely under 1 kPa conditions (Fig. 6A and H). Among the upregulated transcripts, 123 autophagy-related genes were predominantly induced following 24 h of serum starvation (Fig. 7A), whereas 68 genes involved in extracellular matrix organization and remodeling were specifically elevated under 1 kPa conditions (Fig. 7B). Genes selectively downregulated after 24 h of serum starvation were enriched in pathways related to RNA biogenesis and translational control (207 hits, Fig. 7C), while 14 genes associated with actin organization showed reduced expression in response to 1 kPa only (Fig. 7D and E).

Together, our comparative analysis reveals a common transcriptome signature of cilia- and cell cycle-related genes under both conditions, whereas a stronger representation of autophagy and actin-related components was more specifically associated with serum starvation and soft matrix stiffness, respectively.

### Atg5 is required for ciliary length control but not cilia formation on soft substrates

Autophagy has been shown to be required for ciliogenesis under serum-starved conditions, with deletion of *ATG5* leading to a reduction in both the number of ciliated cells and ciliary length [51]. Although autophagy-related genes were not strongly upregulated in 1 kPa samples, we still observed an increased autophagic flux in comparison to glass (serum-enriched) cells, as estimated using the mCherry-GFP-LC3 reporter (Fig. S10A and S10B). However, this increase in autophagic influx on soft matrices was less compared to the 24 h starved cells on glass (Fig. S10B). These observations led us to ask whether cells cultured on 1 kPa substrates require *ATG5* for ciliogenesis and/or ciliary length control.

**Fig. 8:**
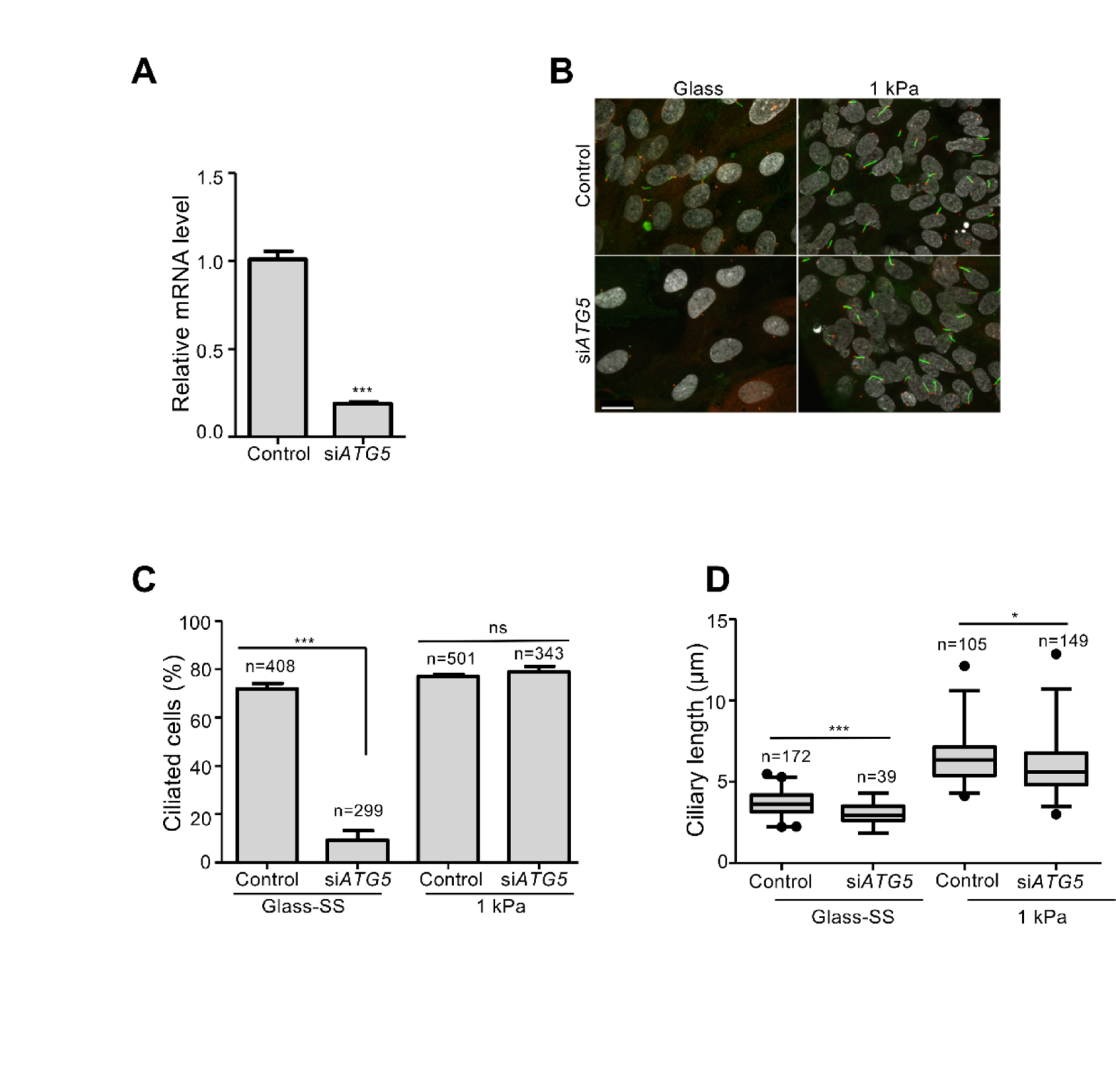
*ATG5* depletion impairs cilia formation in serum-starved cells grown on glass but not in serum-fed cells grown on 1 kPa substrates. (**A**) RPE1 *ARL13B-GFP γ-TUBULIN-mRuby2* cells cultured on glass were treated with mock control or si*ATG5* for 48 h followed by serum-starvation for 48 h in the presence of control or si*ATG5*. The relative *ATG5* mRNA levels were determined by real time PCR. (**B**-**D**) Ciliogenesis was analyzed by the direct fluorescence of Arl13b-GFP and *γ-*Tubulin-mRuby2 in control or si*ATG5*-depleted samples serum-starved on glass (Glass-SS) or seeded on 1 kPa gels in the presence of serum. Representative images are shown in (**B**); γ-Tubulin (red), Arl13b (green), DAPI (grey). Scale bar, 10 μm. Percentage of ciliated cells in the respective conditions are shown in (**C**) and ciliary length measurements are shown in (**D**). Data are from two independent experiments. ***, *p* ≤ 0.001, *, *p* ≤ 0.05 and ns, not significant.

To investigate whether *ATG5* is required for cilia formation under both conditions, we depleted *ATG5* using siRNA (Fig. 8A). *ATG5* depletion in serum-starved cells on glass reduced the proportion of ciliated cells (Fig. 8B and C), and cells that were still able to form cilia displayed shorter cilia (Fig. 8D). Surprisingly, both mock-control and si*ATG5*-treated cells were able to ciliate equally well on 1 kPa substrates, with no significant difference between the two conditions (Fig. 8B and C). However, cilia were significantly shorter in the absence of *ATG5* (Fig. 8D). These data indicate that *ATG5* is required for ciliary length control under both cilia-inducing conditions, but becomes indispensable for cilia formation only upon serum starvation. Collectively, these findings suggest that ciliogenesis induced by serum-starved and soft-substrate conditions may be governed by distinct requirements.

## Discussion

The PC is an evolutionary conserved organelle, and its malfunction in humans is directly associated with a range of genetically inherited ciliopathies^1^. In the past decades, the analysis of cilia formation and length control greatly benefited from studies using cells in culture, in which ciliogenesis can be induced by stimuli that promote cell cycle exit, such as serum starvation, high cell confluency, cell confinement or pharmacological treatment^52,44,43,26^. Interestingly, ECM stiffness has been shown to affect PC behavior in chondrocytes^53^, leading us to ask whether similar stiffness-dependent effects occur in other cell types. Using fibronectin–polyacrylamide hydrogels, we now show that varying ECM stiffness influences ciliogenesis in RPE1 cells under serum-free and serum-rich conditions. We demonstrate that soft (1 kPa) but not stiffer (10 or 100 kPa) substrates significantly induce ciliogenesis in human RPE1 cells independent of serum starvation. Across all stiffness conditions, cilia were longer compared to the glass-control, with 1 kPa cilium being the longest. Notably, longer cilia were observed under both serum-rich and serum-free conditions of soft matrix, indicating that ECM mechanics alone can provide sufficient cues for cilia formation and elongation in RPE1 cells. In chondrocytes, longer cilia were observed with hard (50 kPa) but not soft (5 kPa) substrates under serum-starved conditions^53^, indicating a cell type specific response to matrix stiffness that differs from RPE1 cells. Whether serum-fed chondrocytes ciliate in a matrix-dependent manner remains to be investigated.

Our RNA-seq data suggest that soft matrix inhibits cell proliferation, thereby favoring PC formation. Under serum-rich conditions, RPE1 cells cultured on soft hydrogels (1 kPa) exhibited reduced migration and dense colony formation, consistent with the previous work demonstrating weaker focal adhesions compared to cells on stiff substrates^54,55^. Transcriptome profiling confirmed the downregulation of actin regulators (*ACTR2*, *ACTR3*, *CFL2*), focal adhesion components (*VCL*, *TLN2*), and migration genes (*CORO1A/C*), along with the upregulation of cadherins and protocadherins. Less migration and high cell adherence likely reinforce contact inhibition. As evidenced by reduced Ki-67 and pRb staining, soft hydrogels also induced cell cycle exit, consistent with the fact that cilia formation occurs in the G1/G0 phase of the cell cycle. Notably, RPE1 *CEP83* knockout cells, which are unable to form cilia, also ceased proliferation under soft conditions, indicating that cell cycle arrest is driven by ECM mechanics independent of any inhibitory effect that cilia may exert on the cell cycle.

In agreement with increased ciliogenesis in RPE1 cells grown on 1 kPa substrates in the presence of serum, we found upregulation of multiple ciliary gene categories, including intraflagellar transport (*IFT57*, *IFT140*, *IFT172*), BBSome components (*BBS2*, *BBS4*, *TTC8*), transition zone proteins (*TCTN2*, *TMEM67*), and vesicle trafficking factors (*RAB17*, *EHD3*). Intriguingly, strong transcriptional upregulation of dynein arm and CFAP genes, typically associated with motile cilia, was also observed. Given that dynein-2 (*WDR34*/*WDR60*) is essential for retrograde transport in primary cilia^56^, these findings may suggest broader roles for motility-associated proteins. In addition, two septins, *SEPT4* and *SEPT9*, were also upregulated. While Septin-9 is known to modulate PC organization^57,58^, the role of Septin-4 in ciliogenesis remains unexplored.

Our findings also suggest that ECM stiffness and cilia formation may be coupled through YAP/TAZ mechano-regulation. Inactivation of YAP/TAZ, reflected by its cytoplasmic accumulation, has been shown to facilitate cilia formation in RPE1 cells under high cell confluency conditions^44^. We found that YAP localization remained cytoplasmic in RPE1 cells grown on 1 kPa hydrogels in the presence of serum, consistent with the established mechanical inactivation of YAP/TAZ on soft substrates^59,40,60^. By contrast, under serum-starved conditions on uncoated glass, the YAP nuclear to cytoplasmic ratio was higher than under the 1 kPa condition, but still remained within the previously described non-nuclear/ciliogenic-permissive range^44^. One mechanism by which YAP/TAZ inactivation promotes ciliogenesis is through the downregulation of Aurora kinase A (*AURKA*) and polo-like kinase 1 (*PLK1*), two kinases reported to promote cilia disassembly^44,10^. In agreement with this, our RNA-seq data showed decreased levels of *AURKA* and *PLK1* mRNA in RPE1 cells grown on soft substrates. In addition, we found that YAP/TAZ target genes, including *ANKRD1*, *AMOTL2*, *AXL*, *BCL2*, and *AREG*, were downregulated, further supporting that YAP/TAZ is less active on soft substrates.

We found that not only ciliary number, but also ciliary length increased in RPE1 cells on 1 kPa soft hydrogels in the presence of serum. In addition, these cilia displayed longer distal segments. The altered cytoskeletal organization observed on soft substrates could contribute to this phenotype, as pharmacological depolymerization of actin or microtubules also increased the distal segment ratio^18^. However, because actin and tubulin depolymerization further elongated cilia on soft substrates, cytoskeletal changes alone may not fully explain ciliary lengthening on soft hydrogels. In accordance with our RNA-seq analysis, which revealed increased transcription of transport-related ciliary components, we reasoned that enhanced trafficking to the centrosome contributes to the formation of longer cilia under these conditions. As expected^61^, cilia formation on 1 kPa substrates with serum was IFT88-dependent, in line with intraflagellar transport contributing to cilia assembly on soft matrices. Interestingly, the longer cilia formed on 1 kPa substrates were still competent for SHH signaling, implying that these elongated cilia remain stable and functional.

Transcriptome comparison of soft substrate (serum-fed) with serum-starved RPE1 cells (grown on uncoated glass) highlighted both shared and distinct regulatory programs. Both conditions suppressed cell cycle genes and upregulated ciliary modules associated with PC formation. However, serum starvation uniquely induced autophagy, a process directly related to ciliogenesis^51^, whereas soft substrate specifically downregulated actin filament organization genes and remodeled ECM components. More generally, these broad transcriptomic changes may reflect the overall cellular response to the respective inducing conditions rather than ciliogenesis-specific mechanisms alone. Interestingly, our analysis of *ATG5* suggests that serum starvation- and 1 kPa-induced ciliogenesis may rely on distinct regulatory requirements, as *ATG5* depletion impaired cilia formation upon serum starvation but not on 1 kPa substrates. One possibility is that Atg5-independent autophagy mechanisms may contribute to ciliogenesis on soft substrates^62^. In addition, autophagy has been shown to remove negative regulators of ciliogenesis, including Ofd1, CP110, and Myh9^63,51,64^. However, alternative protein-degradation pathways, such as ubiquitin-dependent proteasomal degradation, are also involved in CP110 removal from basal bodies^65^. These mechanisms may therefore have a greater relative contribution to ciliogenesis on 1 kPa substrates. Together, these observations raise the question of whether and to what extent mechanical and biochemical cues promote ciliogenesis through partially overlapping but mechanistically distinct pathways. Addressing this question in molecular detail will be important in future studies.

Transcriptome analysis revealed upregulation of downstream components of MAP kinase, NF-κB, TGF-β, and SHH signaling pathways in cells grown on soft hydrogels in the presence of serum, representing PC-dependent signaling outputs. Accordingly, we demonstrated that cilia formed on 1 kPa substrates are proficient in activating SHH signaling, further supporting the functionality of cilia formed in RPE1 cells on soft substrates. These results support the concept that ECM mechanics can modulate cilium-dependent signaling outputs, potentially influencing pathways central to development and disease. Interestingly, *GLI1* mRNA levels were elevated under the 1 kPa condition in the absence of SAG, despite the lack of detectable Smo accumulation in primary cilia. Our RNA-seq data also showed increased *GLI1* mRNA levels in 24 h serum-starved ciliated cells (log2fold change, 1.7 and *p* value, 1.15 E -28), indicating that elevated *GLI1* expression is not unique to the soft matrix condition. It is currently unclear why *GLI1* mRNA levels increase in the absence of robust detectable SHH activation. Because the percentage of ciliated cells increases under both conditions, one possibility is that a low level of ciliary SHH activity remains below the detection limit of our ciliary Smo detection assay. Alternatively, *GLI1* expression may be regulated by non-canonical inputs that act independently of ciliary SHH, as reported in other systems, where pathways such as TGF-β and WNT control *GLI1* mRNA levels^66,67^.

We propose that understanding how ciliogenesis is influenced by ECM stiffness will be highly relevant in both tissue- and disease-related contexts. Our data, indicating that RPE1 cells are more prone to ciliate on soft substrates, suggest that tissue-specific ECM stiffness likely contributes to variability in PC abundance and morphology across organs. In the retina, cilia are critical for tissue maintenance and function, as mutations in cilia-related genes contribute to retinal degeneration^68^. Interestingly, ECM stiffening is a hallmark of age-related macular degeneration^69^, raising the question of whether changes in the ECM might lead to cilia loss even in the absence of ciliary mutations. In this context, modulation of matrix mechanics may represent a therapeutic strategy to influence cilia behavior and prevent disease progression. Given the genetic heterogeneity of ciliopathies^70,71^, targeting ECM stiffness could also provide an alternative to gene-based interventions.

## Materials and methods

### Reagents and antibodies

Ammonium-peroxodisulfate (APS) and tetramethylethylenediamine (TEMED) were purchased from Bio-Rad (Hercules, CA, USA). Vinyl trimethoxysilane 98% was obtained from VWR (Wayne, PA, USA). Polydimethylsiloxane (PDMS, Sylgard 184) was purchased from Dow Corning (Midland, MI, USA). Smoothened agonist, SAG was purchased from TOCRIS. Nocodazole and Cytochalasin D were purchased from Sigma-Aldrich. List of primary antibodies, their providers and dilutions are available in supplementary table S1. Secondary antibodies were goat anti-mouse Alexa flour (AF) 647, goat anti-mouse AF 594 and goat anti-rabbit AF 488 (Thermo-Fisher Scientific) and goat anti mouse STAR635P (Abberior). A dilution of 1:500 was used for all the secondary antibodies. Actin was stained with phalloidin-AF 488 (Thermo-Fischer Scientific and DNA was stained with DAPI (4´,6-diamidino-2-phenylindole) (Sigma-Aldrich).

### Polyacrylamide substrates

Before the polymerization of acrylamide, cover glasses with a diameter of 28 and 20 mm were cleaned using a modified RCA cleaning method^72^. To covalently bind the polyacrylamide gels, the glass coverslips with a diameter of 28 mm were silanized using 5% vinyltrimethoxysilan in toluene. The glass slides with a diameter of 20 mm were hydrophilized under a UV/ozone cleaner. The 1, 10 and 100 kPa polyacrylamide hydrogels were fabricated by dissolving 5%, 10% and 20% w/v acrylamide monomer and 0.030%, 0.100% and 0.375% w/v bis-acrylamide crosslinker, respectively in distilled water. Polymerization of the prepared monomer solutions was initiated by adding TEMED (0.3%, v/v) and APS (1 mol%). A 25 µl portion of the monomer-TEMED-APS solution was deposited on the vinyl-silanized glass and sandwiched with the ozone cleaned cover glass. For polymerization, the glass slides were incubated for 15 min before the ozone cleaned glass slide was removed. To remove TEMED and unreacted monomers, the sample was soaked in a mixed solvent of water/DMSO (1/1, v/v) for 1 day and in distilled water for 2 days. The glass with the resulting polyacrylamide hydrogel was glued to the bottom of a petri dish with a central hole using PDMS.

To functionalize the surface of the hydrogel, Sulfo-SANPAH (Sulfosuccinimidyl 6-(4′-azido-2′-nitrophenylamino) hexanoate was used as a crosslinker^73^. Fibronectin (Sigma-Aldrich) was used at a concentration of 200 µg/ml and incubated for 2 h at room temperature. For control, plain glass coverslips coated with 30 µg/ml fibronectin were used.

### Atomic force microscopy

The elasticity (Young’s modulus) of the polyacrylamide gels was measured using an atomic force microscope (NanoWizard, JPK Instruments, Berlin, Germany) and a colloidal probe cantilever (CP-qp-CONT-BSG-B, NanoAndMore, Wetzlar, Germany) with a diameter of 10 μm and a spring constant of *k* = 0.08 N m^− 1^– 0.15 N m^− 1^^74^.

### Cell culture and treatments

Cells lines used in this study were: telomerase immortalized human retinal pigment epithelial (RPE1) cells (ATCC, CRL-4000), RPE1 *CEP83* KO (lab´s stock) and RPE1 cells stably expressing Tet3G or Tet3G *ARL13B-GFP* and *γ-TUBULIN-mRuby2*^18^. RPE1 cells stably expressing mCherry-GFP-LC3 were a kind gift of Jon Lane (University of Bristol, UK)^75^. Cells were cultured in Dulbecco´s Modified Eagle Medium F12 (DMEM F12, Sigma-Aldrich) supplemented with 10% fetal bovine serum (FBS, Sigma-Aldrich), 2 mM L-glutamine (Thermo-Fisher Scientific) and 0.348% sodium bicarbonate (Sigma Aldrich). All cells were cultured at 37 °C and 5% CO_2_.

For drug treatment, cells were seeded at 1 × 10^5^ cells/ml on polyacrylamide-fibronectin samples or glass-control (fibronectin coated glass). After 24 h of seeding, the medium was exchanged to serum-free medium (serum starvation) and cells were incubated for 24 h. Cells were treated with cytochalasin D (CytoD: 200 nM) and Nocodazole (Noco: 100 nM) for 3 h prior to inspection.

For SHH activation on soft matrix and glass-control (fibronectin coated glass), cells were cultured in the presence of serum. After 24 h of seeding, Smoothened (Smo) agonist, SAG (200 nM) was added for 24 h under serum-rich conditions. To analyze Smo accumulation in the cilium under serum deprived condition, cells cultured on glass were serum starved for 24 h then 200 nM of SAG was added for 24 h while keeping the cells under serum-free conditions.

For pRb staining, RPE1-Tet3G cells were used. To assess the influence of cilia on the proliferation potential of soft substrate cells, RPE1-Tet3G wild type and RPE1-Tet3G *CEP83* KO cells were kept in serum-rich media for 4 days before inspection. To visualize actin filaments on hydrogels and glass-control, RPE1 cells cultured under nutrient rich conditions were stained with phalloidin-AF 488 (Thermo-Fischer scientific) according to the manufacturer´s protocol. Similarly, to visualize actin filaments on serum-starved cells, cells cultured on glass under serum-free conditions (48 h) were stained with phalloidin-AF 488 according to the manufacturer´s guidelines.

### siRNA depletion experiments

To deplete *IFT88 or ATG5*, RPE1 *ARL13B-GFP γ-Tubulin mRuby2* cells were cultured (1.5 × 10^5^ cells/ml) on glass-control or 1 kPa sample in serum-rich conditions and treated with human si*IFT88* (20 nM), si*ATG5* (50 nM) or non-targeting siRNA control for 48 h using Opti-MEM (1X) + GlutaMax (Gibco) and lipofectamine RNAimax (Thermo Fischer Scientific). The siRNA-sequences are provided in supplementary table S2.

### Immunofluorescence microscopy

For all the hydrogel experiments, cells were seeded (1 × 10^5^ cells/ml) on fibronectin-coated hydrogels and fibronectin-coated glass coverslips (control). For the immunofluorescence experiments on uncoated glass (referred as glass in the text), cells (30,000/ml) were seeded in 24 well plate. In serum starvation experiments, the medium was exchanged to serum-free medium 24 h after seeding and cells were serum starved for the indicated time period. Cells were fixed with 3% PFA at room temperature for 20 min, quenched with 30 mM glycine (pH 7.5) for 5 min at room temperature (RT) and permeabilized with 0.1% Triton X-100/PBS for 5 min at RT. Samples were stained with the indicated primary and secondary antibodies and DAPI (final concentration of 1 µg/ml). RPE1 mCherry-GFP-LC3 reporter cells were fixed and analyzed by direct fluorescence. Images were acquired as Z-stacks using Plan Apo IR 60x NA 1.27 water immersion objective of laser scanning Nikon confocal (A1r) microscope, equipped with Nikon NIS element acquisition software.

The YAP nuclear to cytoplasmic ratio was quantified by measuring the mean fluorescence intensity of YAP within 5 × 5 pixel areas inside and outside the nucleus (identified by DAPI staining) using maximum-projection images in ImageJ.

### RNA isolation

For bulk RNA-seq analysis, RPE1 *ARL13B-GFP γ-TUBULIN-mRuby2* cells were seeded (1 × 10^5^ cells/ml) on 1 kPa hydrogel-fibronectin samples (35 mm dishes) and glass- control (35 mm dishes coated with fibronectin only) under normal growth/serum-rich conditions. For the RNA-seq of serum-starved cells, uncoated dishes were used, and cells were starved for 6 h and 24 h, whereas 0 h (cycling control) samples were used as their respective controls. For the isolation of RNA, cells were lysed and mRNA isolation was carried out using NucleoSpin^®^ RNA Plus (Macherey-Nagel) kit according to the manufacturer’s protocol.

### Bulk RNA-seq analysis

Paired end RNA sequencing was carried out for 1 kPa (serum-fed) and 24 h serum-starved samples, whereas, single-end sequencing was performed for 6 h serum-starved samples with their respective controls at NGS core facility of the German Cancer Research Centre (DKFZ), Heidelberg, Germany. The quality of the raw data was investigated using FastQC^76^. The adapters were removed with Trimmomatic 0.39^77^. Finally, the reads were mapped with STAR^78^ against the human genome hg38 with an annotation file from GENCODE version 21. The obtained BAM files were sorted using a SAMtools^79^ and counted by the QoRTs^80^.

Principal component analysis, dispersion plot and MA plots were plotted in R. Differential expression analysis was performed using DESeq2 R package under standard conditions^81^. The *p value* and log2fold change were set at < 0.01 and > 0.25 for upregulated genes and < 0.01 and < 0 for downregulated genes respectively. Heatmaps were plotted in R using pheatmap package. Gene ontology (GO) analyses (org.Hs.eg.db) for biological processes (BP), cellular component (CC) and molecular function (MF) were done in R. Stringdb (version:12.0) was used to annotate the selected GO terms.

### cDNA synthesis and real time (RT) PCR

For cDNA synthesis, 1 µg of RNA was reverse transcribed using SensiFAST™ cDNA Synthesis Kit (Bioline) according to the manufacturer’s protocol. Then, 100 ng of cDNA was amplified by real time PCR using SensiFAST™ SYBR Lo-ROX Kit (Bioline). Primer sequences are indicated in supplementary table S3. GAPDH was used as a normalization control in the analysis and fold change was calculated using the ∆∆CT method^82^.

### Statistical analysis and image processing

Statistical analysis was done in graphpad prism and *p value*s were calculated using two tailed Mann Whitney test. *p* < 0.05 was considered significant. All the bar charts represent mean ± standard error of mean (SEM). For all the percentage bar charts, percentages from the individual images were plotted to make the graph. The box of the box and whisker plots represents first quartile, median and third quartile, whereas; whiskers represent 2.5th and 97.5th percentiles and outliers are defined by circles. Nuclear area, nuclear perimeter and ciliary lengths were measured manually in Fiji ImageJ^83^ from the maximum intensity projection images. To measure nuclear volume, Z-stack images were considered and volume measurements were done in ImageJ using 3D objects counter tool. For the analysis of autophagic flux, a custom macro in FIJI was used to quantify the number of red and yellow (GFP^+^ mCherry^+^) dots. The pipeline segmented green and red dots by thresholding with the Yen algorithm. The binary images were later processed with Despeckle and Watershed for puncta detection. The specific regions of interest were then overlapped and quantified.

## Supplementary Information

Below is the link to the electronic supplementary material.

Supplementary Material 1

Supplementary Material 2

Supplementary Material 3

## Data Availability

The RNA-seq datasets generated in the current study have been deposited in the GEO repository under the accession code (GSE336907) (https://www.ncbi.nlm.nih.gov/geo/query/acc.cgi? acc=GSE336907). Additional data are available as supplementary source data accompanying this manuscript or from the corresponding author upon request.
