# Supplementary material for: Soft matrix promotes ciliogenesis in human retinal pigment epithelial cells
Source: Sci Rep. 2026 Jul 13;16:21859. doi: 10.1038/s41598-026-61461-2 (PMC13365419; doi:10.1038/s41598-026-61461-2)
Supplement: Supplementary file 1 — Supplementary Material 1 [file 41598_2026_61461_MOESM1_ESM.docx]

**Supplementary Table 1. Antibodies used in this study.**

| **Primary antibodies** | **Species** | **Dilution** | **Providers** |
| --- | --- | --- | --- |
| YAP (anti-YAP 63.7) | mouse | 1:100 | Santa Cruz, sc-101199 |
| Human Ki-67 | mouse | 1:500 | BD Bioscience # 550609 |
| Smoothened (Smo) | mouse | 1:100 | Santa Cruz, sc-166685 |
| Polyglutamylated Tubulin (GT335) | mouse | 1:1000 | Adipogen, AG-20B-0020 |
| Phospho retinoblastoma (pRb) | rabbit | 1:200 | Cell signaling #8516 |
| α-Tubulin | mouse | 1:1000 | Sigma (TAT-1), AB_10013740 |
| Arl13b | rabbit | 1:500 | Acris # 17711-1-AP |

**Supplementary Table 2. siRNA used in this study.**

| **Gene** | **Company** | **siRNA-sequence (5´-3´)** |
| --- | --- | --- |
| Non-targeting control (smart pool) | Dharmacon | UGGUUUACAUGUCGACUAA  UGGUUUACAUGUUGUGUGA  UGGUUUACAUGUUUUCUGA  UGGUUUACAUGUUUUCCUA |
| Human IFT88 | Ambion | ACUAAGUGCCAGACUCAUUTT |
| Human ATG5 (smart pool) | Dharmacon | GAUAUAGGGCAUAGGAUUA  AGAUAAAGUUGGUCAAAGA  GGAUAUAGGGCAUAGGAUU  CCAACAGAUUGAAGGAUCA |

| **Gene** | **Forward primer (5´-3´)** | **Reverse primer (5´-3´)** |
| --- | --- | --- |
| DNAH7 | TGACCCCAGCACCAGAAATC | CCTGCTGAACGAGACTGTGT |
| DNAAF11 | CAGCTTGTCCTTCCTGCAGA | GTTCTCTGCTCCTGTCCGAG |
| CFAP69 | TGTCATGCCAGGGAAATGCT | ACTGTAACGCATCTGGGCAA |
| TTC29 | TGGGAAGAAAGAAGCCGAGG | CATATCCGCCCCTGTGTCAA |
| IFT140 | AGTCAAAGGTCTTCTGGCCG | GCGTGATGTTTCCTTGGAGC |
| IFT70A | CCCTGATCACTTGCCAGACA | ACTTGCTTGGTGAGTCTCCG |
| SEPT4 | TGGGGATGCAGTCAACAACA | TTTCGGTTCAGGCCACTCTC |
| ANKRD1 | TCAGAGCCTTCCCACCCATA | ACATGCCTTCCCTTGCTTCA |
| ADAMTS14 | AGTACCAGGTCTGCAACAGC | GTCATCGTCAGGCTCGTAGG |
| PCDHGB7 | CGGTAGAGTGCACTTTCTCCA | AGCGTGGGGTAGAACAAAGG |
| PCDHGC5 | GCTGTGCATGTTGTCCTTGT | TCCTGAGCAACATTCCCCAC |
| GLI1 | GATGACCCCACCACCAATCAGTAG | AGACAGTCCTTCTGTCCCCACA |
| ATG5 | TCATCCCACAGCCAACAGAT | GCCTCCACCAAACCTGATTG |
| GAPDH | GATTTGGTCGTATTGGGCGC | TTCCCGTTCTCAGCCTTGAC |

**Supplementary Table 3. Primers used in this study.**
