# Supplementary figures and images for: Soft matrix promotes ciliogenesis in human retinal pigment epithelial cells
Source: Sci Rep. 2026 Jul 13;16:21859. doi: 10.1038/s41598-026-61461-2 (PMC13365419; doi:10.1038/s41598-026-61461-2)

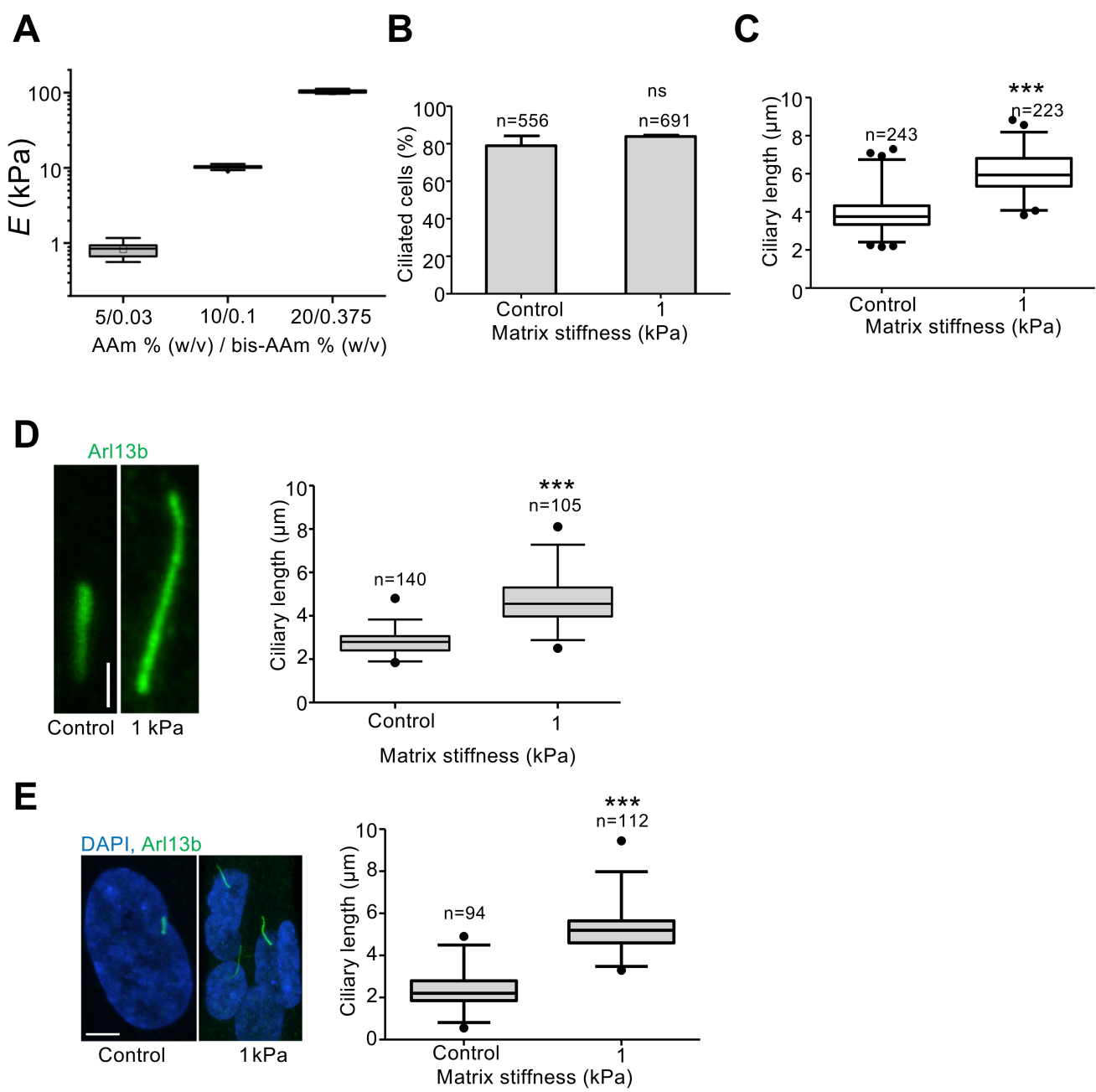

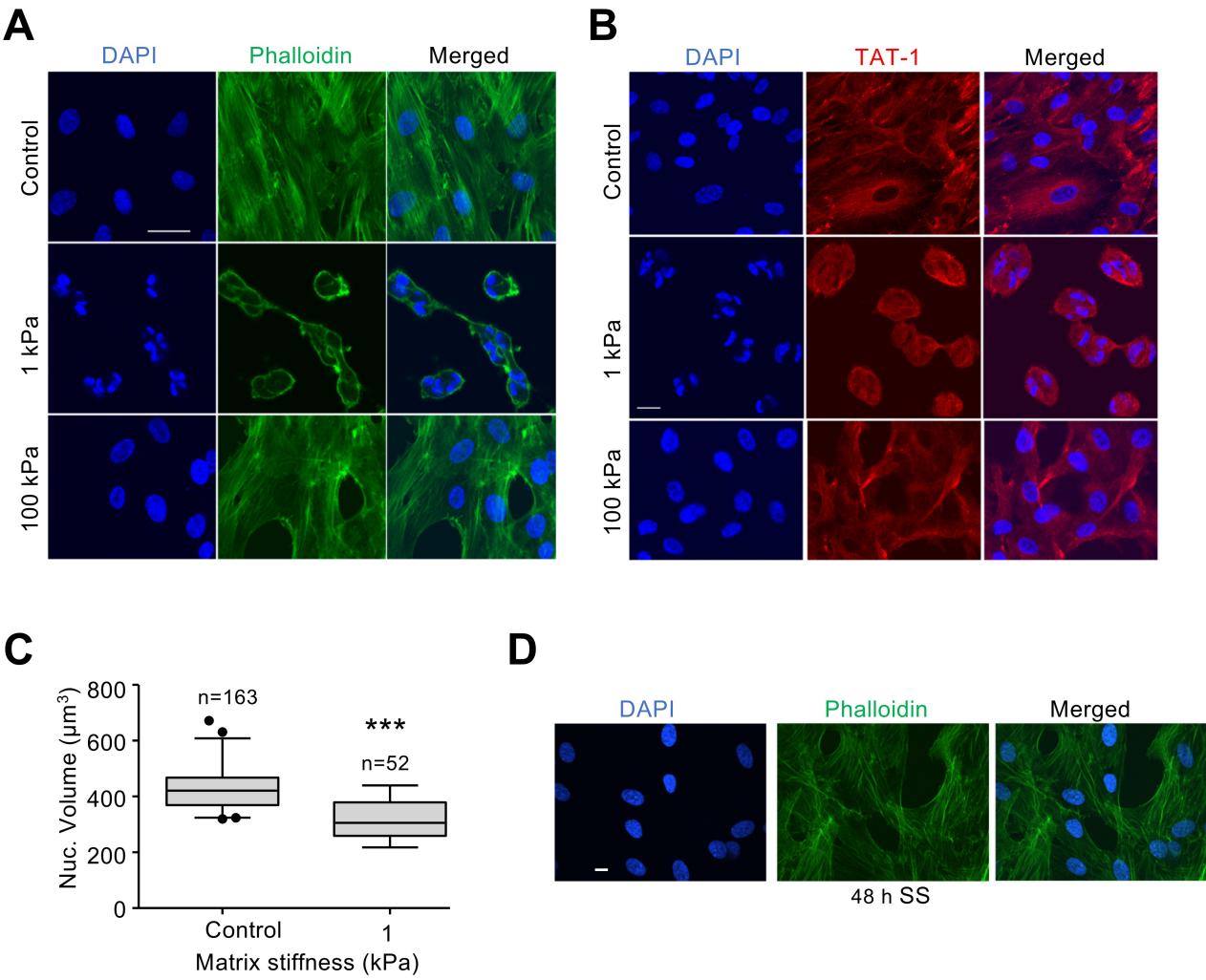

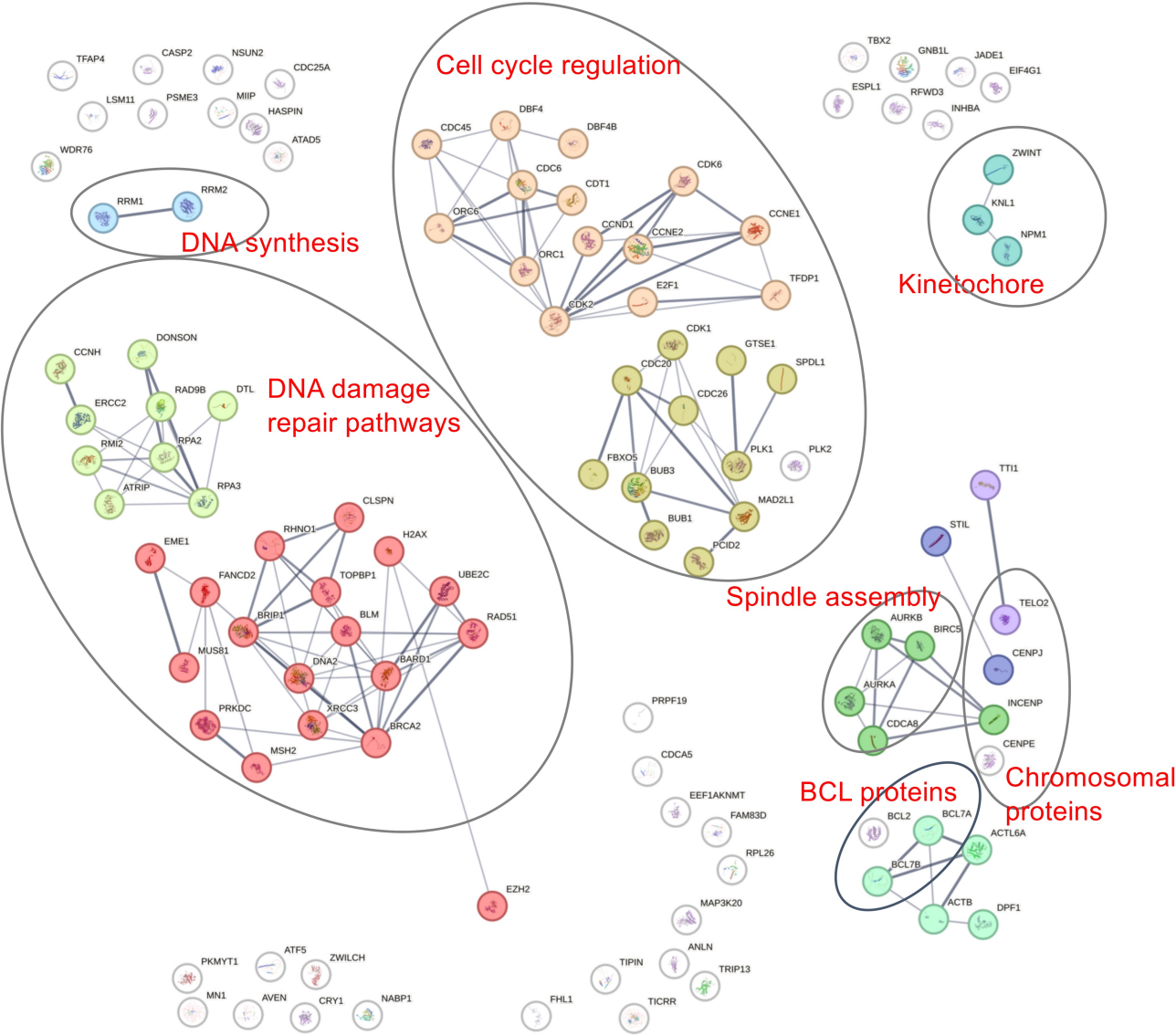

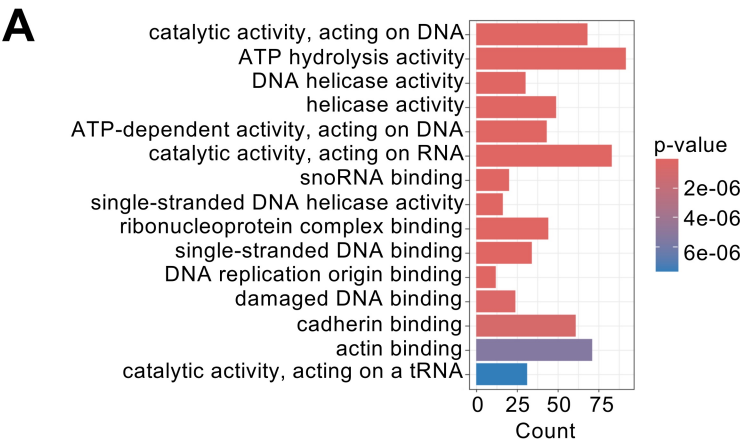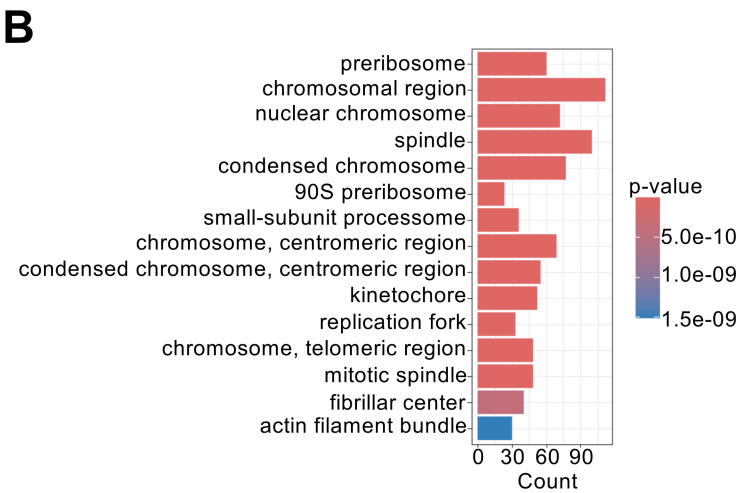

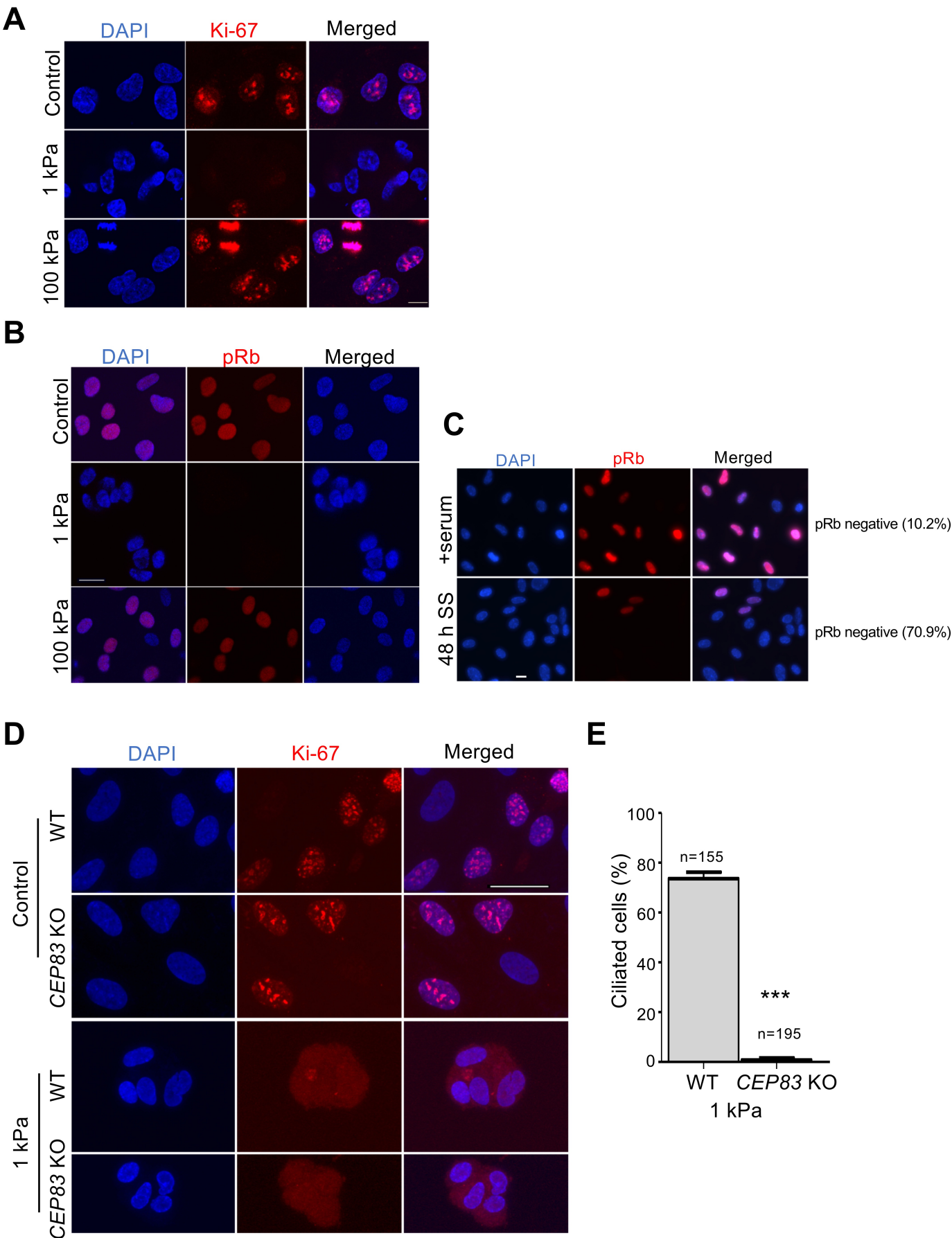

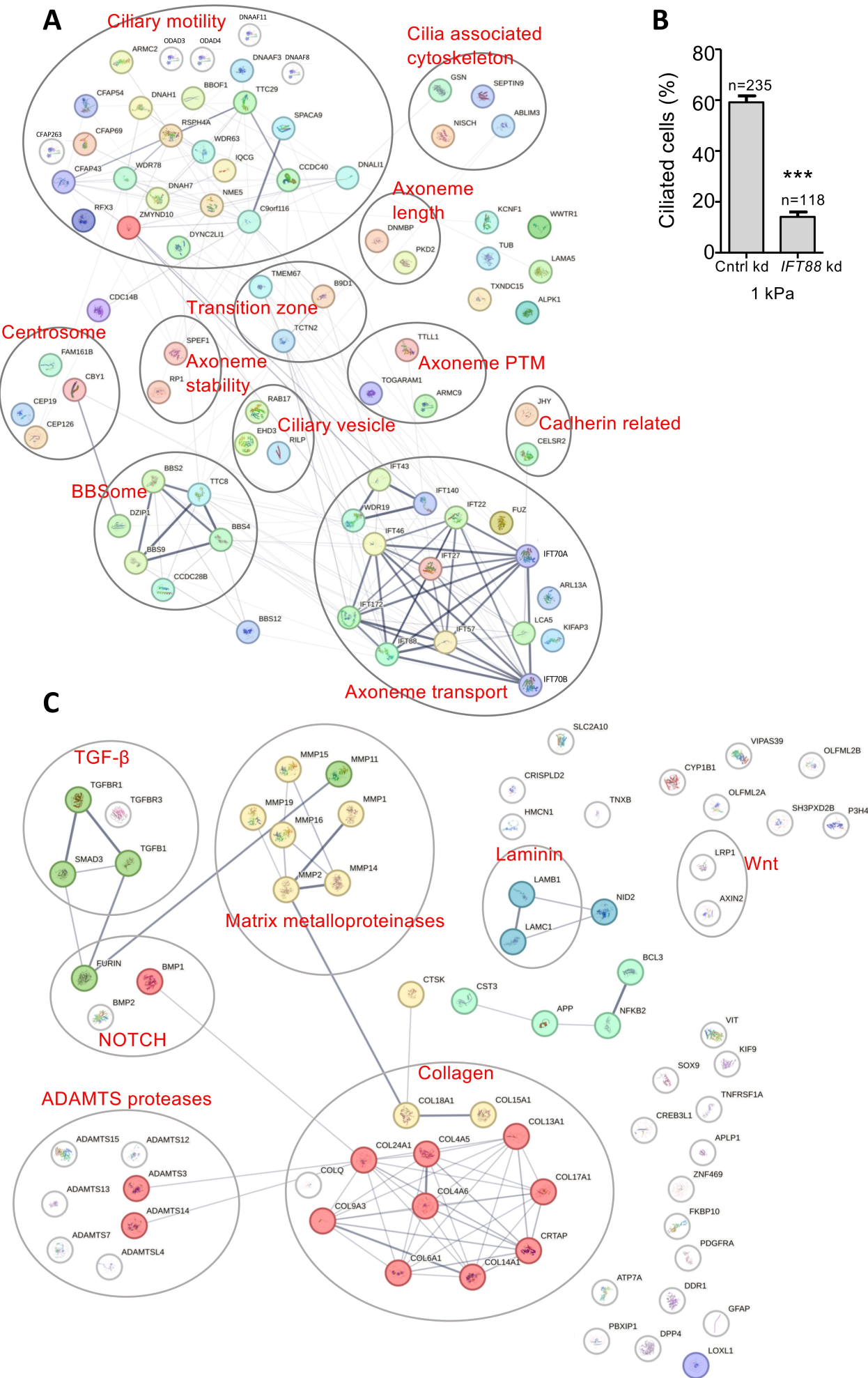

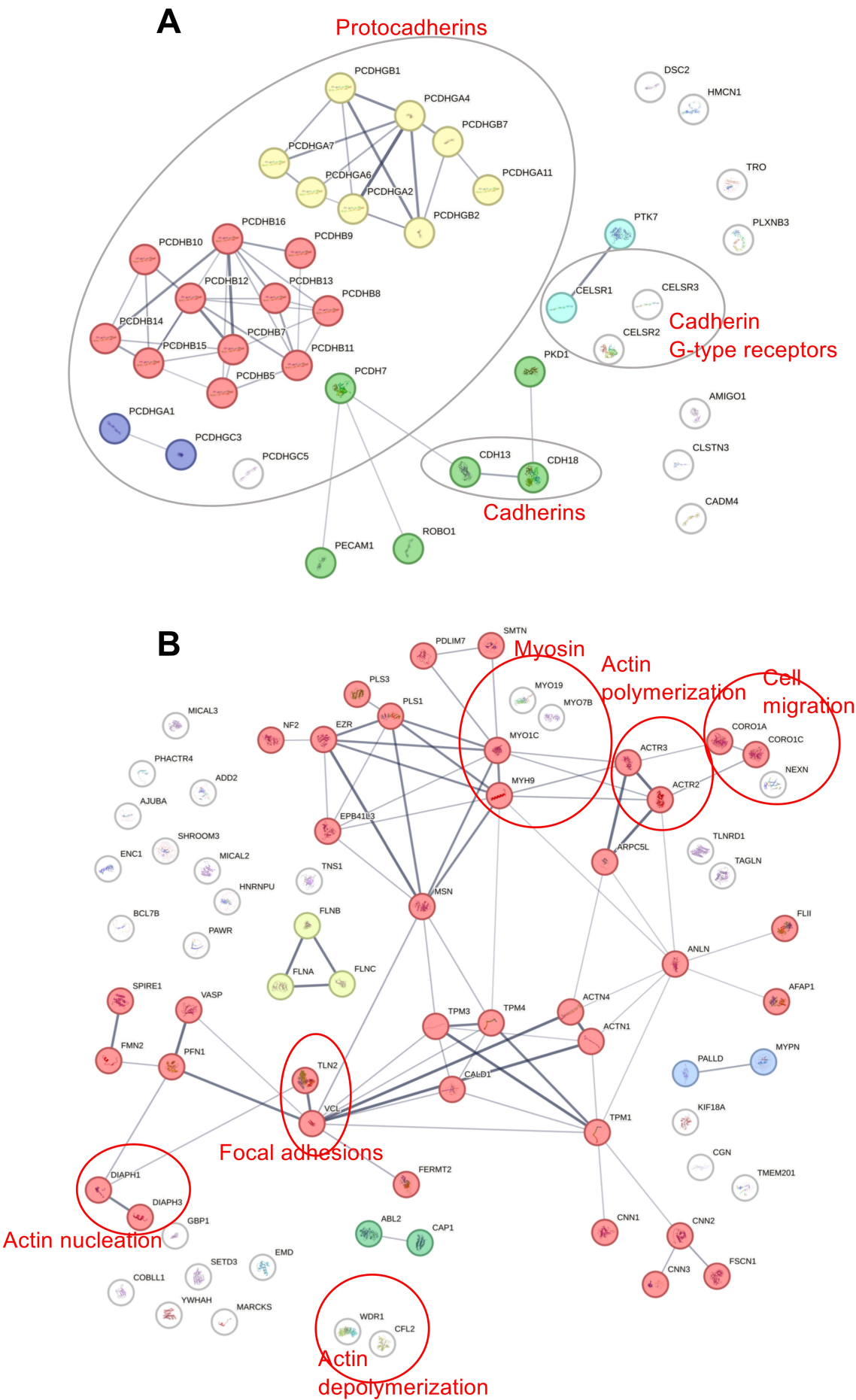

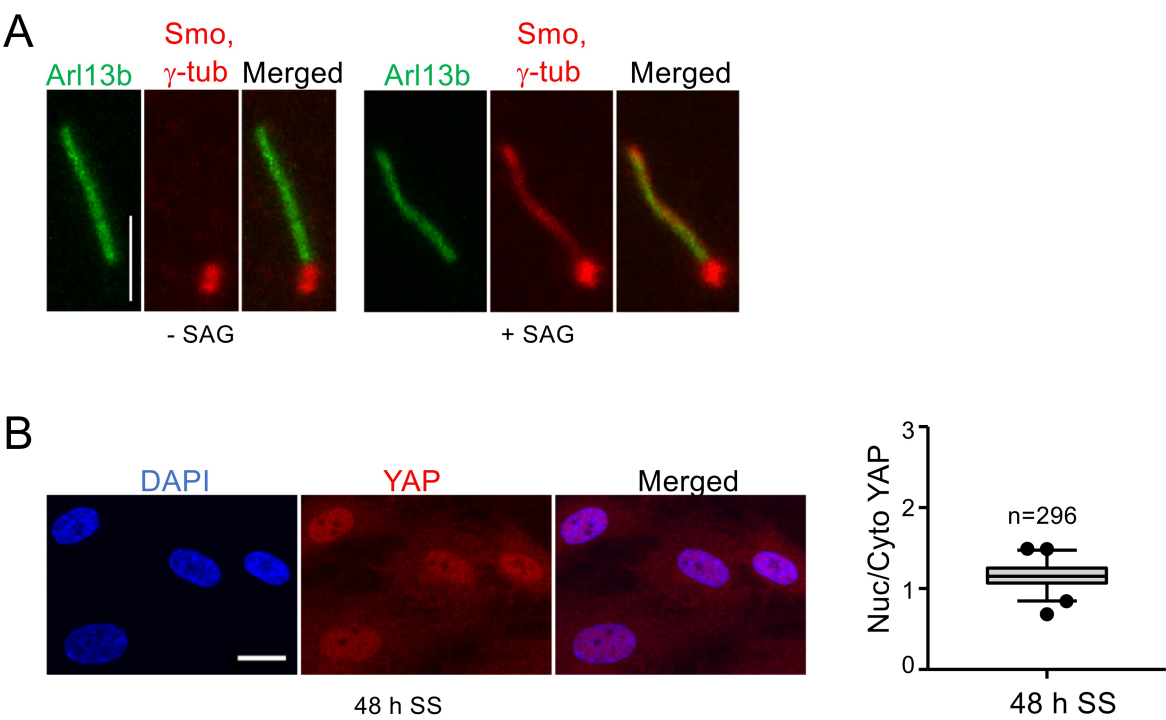

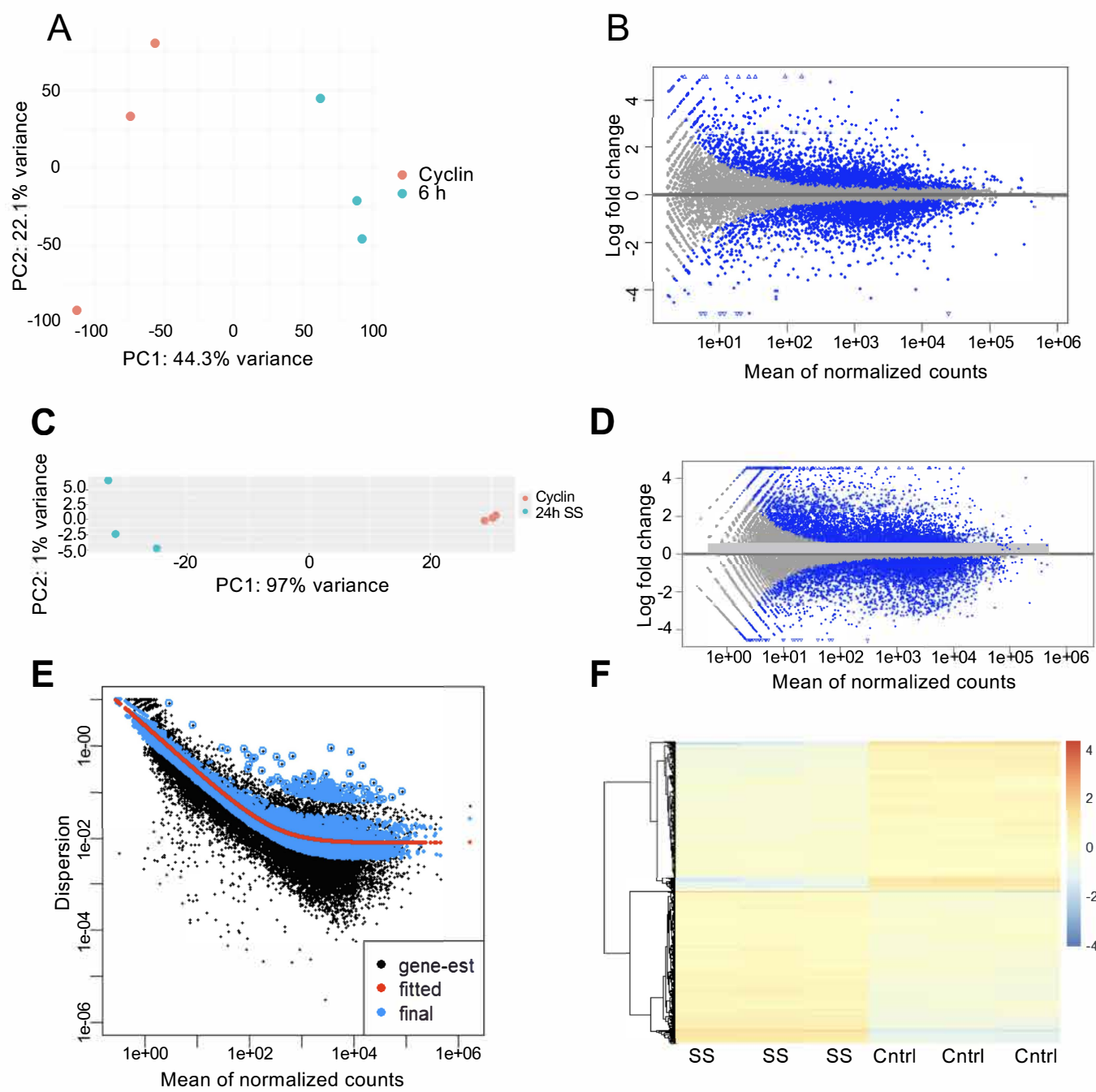

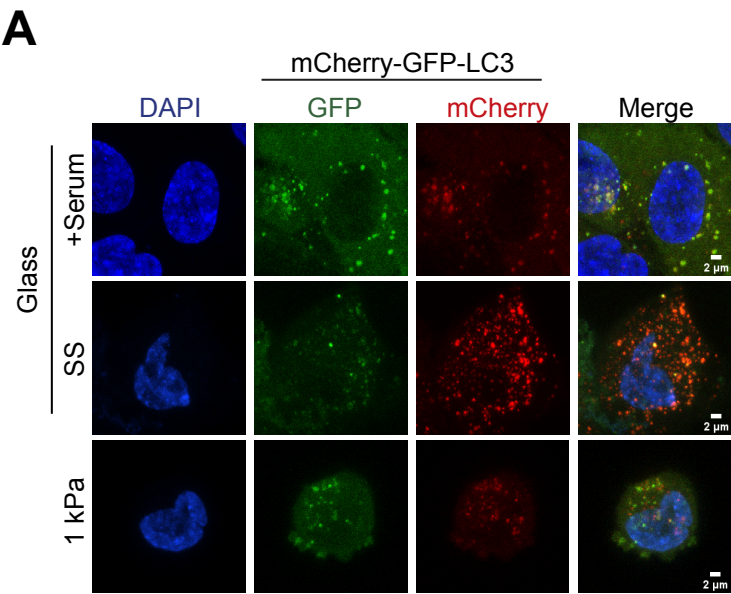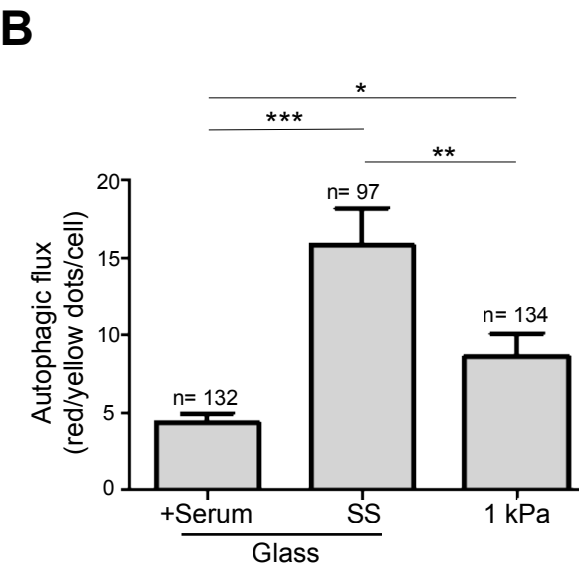

Supplement: Supplementary file 2 — Supplementary Material 2 [file 41598_2026_61461_MOESM2_ESM.pdf]
