# Supplementary material for: Soft matrix promotes ciliogenesis in human retinal pigment epithelial cells
Source: Sci Rep. 2026 Jul 13;16:21859. doi: 10.1038/s41598-026-61461-2 (PMC13365419; doi:10.1038/s41598-026-61461-2)
Supplement: Supplementary file 3 — Supplementary Material 3 [file 41598_2026_61461_MOESM3_ESM.docx]

**Supplementary Figure Legends**

**Figure S1. Soft substrate increases PC length in RPE1 cells upon serum depletion.**

**(A)** Elasticity measurement of polyacrylamide hydrogels by AFM nanoindentation. Three different compositions with varying amounts of acrylamide monomer (AAm) and bis-acrylamide (bis-AAm) crosslinker were subjected to analysis. Measurements were carried out in PBS at room temperature. For each hydrogel substrate, at least 31 force curves were quantified. **(B-C)** RPE1 ARL13B-GFP γ-TUBULIN-mRuby2 cells were cultured on 1 kPa or glass-control conditions and serum starved for 24 h before inspection. The percentage of ciliated cells (B) and the length of cilia (C) are shown. Data are from two independent biological replicates. **(D)** RPE1 wild type (WT) cells were cultured on 1 kPa and glass-control in the presence of serum. After 24 h of seeding, cells were serum starved for 48 h followed by staining with Arl13b antibodies (ciliary membrane marker) and DAPI before inspection. Representative images of primary cilium (left) and ciliary length graphs (right) are shown. Data are from two replicates. Scale bar, 2 μm. (E) RPE1 wild type (WT) cells cultured on 1 kPa and glass-control in the presence of serum for 24 h were stained for Arl13b and DAPI. Representative images (left) and ciliary length graphs (right) are shown. Data are from two replicates. Scale bar, 8 μm. The number of samples (n) is indicated on the graphs. Statistical analysis according to two tailed Mann Whitney test: ***, *p* ≤ 0.001; ns, non-significant.

**Figure S2. Effects of soft substrates on nuclei morphology and cytoskeleton.**

**(A)** Representative images of RPE1WT cells cultured on glass, 1 kPa and 100 kPa matrices in serum-rich medium for 24 h, stained for actin with Phalloidin AF 488. Scale bar, 25μm **(B)** Representative images of RPE1WT cells cultured on glass, 1 kPa and 100 kPa matrices in serum-rich medium for 24 h, stained for tubulin with TAT-1 antibodies. Scale bar, 25μm. (C) RPE1 *ARL13B-GFP γ-TUBULIN-mRuby2* cells cultured on 1 kPa and glass-control were serum starved for 48 h. DAPI staining was used to mark the nuclei. Z-stack images were obtained, and nuclear volume was measured in ImageJ. Data are from two independent experiments. The total number of nuclei considered for the analysis are mentioned as ´n` on the graph for each condition. Statistical analysis was performed using two tailed Mann Whitney test. ***, *p* ≤ 0.001. (D) RPE1 wild type (WT) cells cultured on glass and serum starved for 48 h were stained with Phalloidin AF 488 according to the manufacturer´s protocol. Nuclei were marked with DAPI staining. Representative images are shown. Scale bar, 20 μm.

**Figure S3. Soft matrix transcriptome illustrates reduced expression of cell cycle regulators.**

Gene ontology term for downregulated genes on 1 kPa (from Fig. 2E), associated with biological processes related to the regulation of cell cycle phase transitions.

**Figure S4. GO enrichment analysis based on molecular function and cellular component for downregulated genes on 1 kPa.**

**(A-B)** GO analysis showing the top categories for downregulated genes on 1 kPa relative to glass-control, sorted based on *p* < 0.01 and log2fold change < 0. GO terms related to molecular function are shown in (A), whereas GO terms representing cellular components are shown in (B).

**Figure S5. Effect of soft matrix (1 kPa) on cell cycle progression.**

**(A)** RPE1 *ARL13B-GFP γ-TUBULIN-mRuby2* cells were cultured under serum-rich conditions and stained for the proliferation marker Ki-67. Representative Ki-67 staining (red) for glass-control, 1 kPa and 100 kPa conditions is shown. Scale bar, 25 μm. **(B)** RPE1-Tet3G cells grown in the presence of serum were stained for phosphorylated Rb (pRb). Representative pRb staining for glass-control, 1 kPa and 100 kPa conditions is shown in red. Scale bar, 25 μm. (C) RPE1 wild type (WT) cells cultured on uncoated glass in the presence and absence of serum (48 h SS) were stained with a proliferation marker, pRb and a nuclear marker, DAPI before inspection. The percentage of pRb negative nuclei are mentioned on the right for both conditions. Scale bar, 20 μm. **(D)** RPE1 wild type (WT) and RPE1 *CEP83* knockout (KO) cells were cultured on glass-control and 1 kPa matrix in serum-rich conditions for 4 days before fixation and staining for Ki-67 (shown in red). Scale bar, 15 μm. **(E)** Percentage of ciliated cells from (D) was determined based on antibody staining of Arl13b (ciliary membrane marker) and γ-Tubulin (basal body marker) in RPE1 wild type (WT) and RPE1 *CEP83* knockout (KO) cells.

DNA was stained with DAPI. Data shown in (A-B) are from three independent biological replicates, whereas data shown in (D-E) are from two independent biological replicates. Sample numbers (n) are indicated on the graph. Statistical analysis according to two tailed Mann Whitney test: ***, *p* ≤ 0.001.

**Figure S6. Effect of soft matrix (1 kPa) on axoneme, BBSome, cilia motility and ECM genes.**

**(A)** GO term for upregulated genes on 1 kPa (from Fig. 2K), associated with biological processes related to cilium assembly and cilium organization. **(B)** RPE1 *ARL13B-GFP γ-TUBULIN-mRuby2* cells cultured on 1 kPa matrix in serum-rich medium were treated with 20 nM of non-targeting siRNA (control, Cntrl kd) or IFT88 siRNA (*IFT88* kd) for 48 h. The graph indicates the percentage of ciliated cells determined by direct fluorescence analysis, with Arl13b-GFP as a ciliary membrane marker and γ-Tubulin-mRuby2 as a basal body marker. Sample numbers (n) are indicated on the graph. Data are from two independent biological replicates. **(C)** Gene ontology term for upregulated genes on 1 kPa (from Fig. 2K), associated with biological processes related to extracellular matrix organization.

**Figure S7. Effect of soft matrix (1 kPa) on adhesion- and actin-associated genes.**

**(A)** GO term for upregulated genes on 1 kPa (from Fig. 2K), associated with biological processes related to homophilic cell adhesion via plasma membrane adhesion molecules. **(B)** GO term for downregulated genes on 1 kPa (from Fig. S4B), associated with cellular components related to actin filament bundle formation.

**Figure S8. YAP localization and Smo cilia accumulation upon serum deprivation.**

**(A)** RPE1 *ARL13B-GFP γ-TUBULIN-mRuby2* cells cultured on glass and serum-starved for 24 h were treated with either 200 nM of SAG (Smothened agonist, + SAG) or water (control, - SAG) for 24 h while keeping the cells under serum-deprived conditions. Cilium was visualized by direct fluorescence microscopy, whereas Smoothened (Smo) entry into the cilium was visualized by immunostaining with Smo antibodies. Representative images are shown. Scale bar, 2.5µm. **(B)** RPE1 *ARL13B-GFP γ-TUBULIN-mRuby2* cells cultured on uncoated glass were serum deprived for 48 h and stained with DAPI and YAP antibody before inspection. Representative images (left) and nuclear/cytoplasmic ratio (right) are shown. Data are from three independent experiments. Sample number `n` is shown on the graph. Scale bar, 20 μm.

**Figure S9. Quality assessment of transcriptomic data for 6 h and 24 h of serum starvation. (A-D)** Bulk RNA-seq was performed on RPE1 cells cultured on glass and serum starved for 6 h (A-B) or 24 h (C-D). PCA plots (A and C) and MA plots of normalized counts and log fold change (B and D) are shown. **(E)** Dispersion plot analysis of transcriptome data for the 24 h serum-starved condition. **(F)** Heatmap of 11,354 differentially expressed genes (*p* < 0.05) for the 24 h serum-starved condition.

**Figure S10. Comparison of autophagy under starvation conditions and soft matrix conditions.**

**(A)** Representative images of RPE1 mCherry-GFP-LC3 cultured on glass in the presence of serum or serum-starved for 24 h (SS) or grown on 1 kPa for 24 h in the presence of serum. Individual channels and merged images are shown. DNA was stained with DAPI. Scale bar, 2 µm. **(B)** Ratio of red/yellow dots per cell are shown for respective conditions. Data are from two independent experiments. Statistical significance is calculated using Mann Whitney test: ***, *p* ≤ 0.001; **, *p* ≤ 0.01; *, *p* ≤ 0.05.
